# Effects of extracellular metabolic acidosis and out-of-equilibrium CO_2_/HCO_3_^−^ solutions on intracellular pH in cultured rat hippocampal neurons

**DOI:** 10.3389/fphys.2024.1434359

**Published:** 2024-10-09

**Authors:** Patrice G. Bouyer, Ahlam I. Salameh, Yuehan Zhou, Tiffany N. Kolba, Walter F. Boron

**Affiliations:** ^1^ Department of Cellular and Molecular Physiology, Yale University School of Medicine, New Haven, CT, United States; ^2^ Department of Biology, Valparaiso University, Valparaiso, IN, United States; ^3^ Preclinical Sciences Division, Kent State University College of Podiatric Medicine, Independence, OH, United States; ^4^ Department of Physiology & Biophysics Case Western Reserve University School of Medicine, Cleveland, OH, United States; ^5^ Department of Mathematics & Statistics, Valparaiso University, Valparaiso, IN, United States

**Keywords:** CO_2_/HCO_3_^−^ out of equilibrium solutions, pH regulation, HCO_3_^−^ sensor pH_o_ sensor, acid base, neuron

## Abstract

Metabolic acidosis (MAc)—an extracellular pH (pH_o_) decrease caused by a [HCO_3_^−^]_o_ decrease at constant [CO_2_]_o_—usually causes intracellular pH (pH_i_) to fall. Here we determine the extent to which the pH_i_ decrease depends on the pH_o_ decrease vs the concomitant [HCO_3_^−^]_o_ decrease. We use rapid-mixing to generate out-of-equilibrium CO_2_/HCO_3_^−^ solutions in which we stabilize [CO_2_]_o_ and [HCO_3_^−^]_o_ while decreasing pH_o_ (pure acidosis, pAc), or stabilize [CO_2_]_o_ and pH_o_ while decreasing [HCO_3_^−^]_o_ (pure metabolic/down, pMet↓). Using the fluorescent dye 2′,7′-bis-2-carboxyethyl)-5(and-6)carboxyfluorescein (BCECF) to monitor pH_i_ in rat hippocampal neurons in primary culture, we find that—in naïve neurons—the pH_i_ decrease caused by MAc is virtually the sum of those caused by pAc (∼70%) + pMet↓ (∼30%). However, if we impose a first challenge (MAc_1_, pAc_1_, or pMet↓_1_), allow the neurons to recover, and then impose a second challenge (MAc_2_, pAc_2_, or pMet↓_2_), we find that pAc/pMet↓ additivity breaks down. In a twin-challenge protocol in which challenge #2 is MAc, the pH_o_ and [HCO_3_^−^]_o_ decreases during challenge #1 must be coincident in order to mimic the effects of MAc_1_ on MAc_2_. Conversely, if challenge #1 is MAc, then the pH_o_ and [HCO_3_^−^]_o_ decreases during challenge #2 must be coincident in order for MAc_1_ to produce its physiological effects during the challenge #2 period. We conclude that the history of challenge #1 (MAc_1_, pAc_1_, or pMet↓_1_)—presumably as detected by one or more acid-base sensors—has a major impact on the pH_i_ response during challenge #2 (MAc_2_, pAc_2_, or pMet↓_2_).

## Introduction

Neuronal activity produces transient shifts in extracellular pH (pH_o_). The direction of these shifts depends on the location in the brain, and their peak magnitude depends on the intensity and duration of the activity (for review, see Chesler, 2003). Conversely, changes in pH_o_ can alter neuronal excitability by modulating pH-sensitive ion channels (Meech and Thomas, 1987; Tang et al., 1990; Church et al., 1998). Moreover, changes in pH_o_ also cause intracellular pH (pH_i_) to shift in the same direction (Ellis and Thomas, 1976; Vaughan-Jones, 1986; Tolkovsky and Richards, 1987; Bouyer et al., 2004) and such changes in pH_i_ also modulate neuronal excitability (Filosa et al., 2002; Wang et al., 2002). In addition to these effects on ion channels, changes in pH_o_ and/or pH_i_ can modulate other components of the machinery of neurotransmission, such as vesicular amine transporters, and transporters that mediate the reuptake of glutamate and serotonin (Keyes and Rudnick, 1982; Kanai et al., 1995; Eiden et al., 2004). Therefore, it is important for a neuron to maintain an appropriate pH_i_ in the face of shifting values of pH_o_. To achieve this task, neurons like other cells, are armed with acid-base transporters (Ruffin et al., 2014). Among these, Na-H exchangers play important roles in maintaining steady-state pH_i_ as well as in extruding acid during the recovery of pH_i_ from an acid-load (Bevensee et al., 1996; Raley-Susman et al., 1991; Tolkovsky and Richards, 1987; Yao et al., 1999). In neurons exposed to CO_2_/HCO_3_^−^, the Na^+^-coupled HCO_3_^−^ transporters—the Na^+^-driven Cl/HCO_3_ exchanger (Boron and De Weer, 1976; Russell and Boron, 1976; Thomas, 1977) and the electroneutral Na/HCO_3_ cotransporters NBCn1 and NBCn2 (Cooper et al., 2005; Parker et al., 2008)—are among the most potent acid extruders (i.e., transporters that mediate the efflux of H^+^ or influx of alkali; see refs. Schwiening and Boron, 1994; Cooper et al., 2005; 2009; Gurnett et al., 2008). In the presence of CO_2_/HCO_3_^−^, the Cl-HCO_3_ exchanger AE3 is a potent acid loader that helps neurons to recover after alkaline loads (Kopito et al., 1989; Raley-Susman et al., 1993; Brett et al., 2002; Hentschke et al., 2006; Svichar et al., 2009; Ruffin et al., 2014; Salameh et al., 2017).

Although the field has witnessed substantial progress in clarifying the molecular nature of the Na-H exchangers (Donowitz et al., 2013) and HCO_3_^−^ transporters (Parker and Boron, 2013; Romero et al., 2013; Thornell et al., 2025) that are involved in pH_i_ homeostasis, the same is not true for understanding how changes in the key extracellular acid-base parameters—pH_o_, [CO_2_]_o_, and [HCO_3_^−^]_o_—modulate these transporters and, thus, pH_i_. Of course, changes in the above parameters could directly affect transporters. For example, a change in pH_o_ would modulate Na-H exchange because H^+^ competes with extracellular Na^+^ (Aronson et al., 1982). Change in pH_o_ or [HCO_3_^−^]_o_ would modulate the Na^+^-coupled HCO_3_^−^ transporters because in at least two cases, and possibly all, the HCO_3_^−^-related substrate is CO_3_^=^ or NaCO_3_^−^ (Lee et al., 2023), the concentrations of which are very sensitive to changes in pH_o_ and [HCO_3_^−^]_o_. One mechanism by which metabolic acidosis (MAc; a decrease in [HCO_3_^−^]_o_ that causes pH to fall) causes pH_i_ in neurons to fall (Bouyer et al., 2004; Salameh et al., 2014) is an enhanced activity of an acid loader, the Cl-HCO_3_ exchanger AE3 (Salameh et al., 2017).

One mechanism for the aforementioned enhanced AE3 activity is the fall in [HCO_3_^−^]_o_ that accompanies MAc. However, other potential mechanisms could involve sensors for pH, CO_2_, or HCO_3_^−^ (Tresguerres et al., 2010) that respond to changes in acid-base status by modulating acid-base transporters. Established acid-base sensors include three G-protein–coupled proteins—GPR68 (aka, OGR1; see (Ludwig et al., 2003; Tomura et al., 2005; Mohebbi et al., 2012), GPR4 (Ludwig et al., 2003; Sun et al., 2010), and GPR65 (aka, TDAG8; see Ishii et al., 2005)—that activate in response to decreases in pH_o_. In addition, the soluble adenylyl cyclase sAC (Chen et al., 2000) and certain receptor guanylyl cyclases (Schulz et al., 1998; Sun et al., 2009) become more active in response to increases in [HCO_3_^−^]_i_; numerous ion channels (e.g., TASK, ASIC, TRPV) respond to changes in pH_o_ or pH_i_ (for reviews, see Montell, 2001; Lesage, 2003 *a*; Holzer, 2009; Tresguerres et al., 2010); and the tyrosine kinase Pyk2 becomes more active in response to decreases in pH_i_ (Li et al., 2004). In *Drosophila*, Gr21a and Gr63a are reported to be CO_2_ receptors; ectopic expression of Gr21a and Gr63a confers chemosensitivity to olfactory neurons, whereas gene deletion prevents behavioral response of *Drosophila* to CO_2_ (Jones et al., 2007). Finally, the receptor protein tyrosine phosphatase γ (RPTPγ) appears to respond oppositely to changes in both [CO_2_]_o_ and [HCO_3_^−^]_o_ but is insensitive to pH_o_ changes (Boedtkjer et al., 2016; Zhou et al., 2016). The review by Thornell et al. (2024) discusses such acid-base sensors.

The development of out-of-equilibrium (OOE) CO_2_/HCO_3_^−^ solutions (Zhao et al., 1995)—which can have virtually any combination of [CO_2_], [HCO_3_^−^], and pH in the pathophysiological range of pH values—makes it possible to ask whether cells have a mechanism for sensing extracellular CO_2_ or HCO_3_^−^ *per se*, independent of pH. Indeed, using OOE solutions, Zhou et al. demonstrated that isolated renal proximal tubules increase their rate of bicarbonate reabsorption (i.e., H^+^ secretion) in response to isolated decreases in basolateral [HCO_3_^−^] (i.e., holding basolateral [CO_2_] and pH constant) or to isolated increases of basolateral [CO_2_], but not to isolated changes in basolateral pH_o_ (Zhou et al., 2005). These results were the first to demonstrate unequivocally that, independent of pH, the two components of the major blood buffer—CO_2_/HCO_3_^−^ —can act as potent modulators of a biological function.

An earlier study of cultured rat neurons showed that the pH_i_ responses to extracellular respiratory acidosis (i.e., an increase in [CO_2_]_o_ that causes pH_o_ to fall), to extracellular respiratory alkalosis (i.e., a decrease in [CO_2_]_o_), or to metabolic alkalosis (i.e., an increase in [HCO_3_^−^]_o_, at fixed [CO_2_]_o_, that causes pH_o_ to rise) were each indistinguishable between medullary-raphé (MR) vs hippocampal (HC) neurons. In all cases, neuronal pH_i_ changed in the same direction as pH_o_, with a steady-state ΔpH_i_/ΔpH_o_ of ∼60%. However, the responses to extracellular metabolic acidosis were not uniform. The majority of the MR neurons and the minority of HC neurons exhibited the expected response to MAc: a reversible pH_i_ decrease and a ΔpH_i_/ΔpH_o_ of ∼65%. In contrast to these “MAc-sensitive” neurons, a minority subpopulation of MR neurons and the majority of HC neurons exhibited a ΔpH_i_/ΔpH_o_ of only ∼9% (Bouyer et al., 2004). Moreover, returning these “MAc-resistant” neurons from the MAc solution to a solution with a normal acid-base status causes pH_i_ to rebound above the initial baseline. An analysis of various possibilities (see the discussion of Bouyer et al., 2004), led to the hypothesis that MAc-resistant MR and HC neurons have an extracellular “HCO_3_^−^ sensor” that can detect decreases in [HCO_3_^−^]_o_ *per se*, and respond with a near-instantaneous increase in the rate of acid extrusion over acid loading, the effect of which is to minimize decreases in pH_i_.

In the present study, we re-examine the effect of MAc on the pH_i_ of rat HC neurons by using OOE technology to break MAc artificially into its two component parts, “pure” acidosis (pAc: ↓pH_o_ at fixed [CO_2_]_o_ and [HCO_3_^−^]_o_) and pure metabolic/down (pMet↓: ↓[HCO_3_^−^]_o_ at fixed [CO_2_]_o_ and pH_o_). We also examined acidosis (Ac) in the nominal absence of CO_2_/HCO_3_^−^. Finally, we performed seven twin-challenge protocols in which we examined the effect on pH_i_ of a challenge (e.g., pMet↓), followed by a recovery period, and then a second challenge (e.g., MAc). Surprisingly, we find that the effects of pAc and pMet↓ do not necessarily sum, and that neurons often respond to pMet↓ by with a paradoxical pH_i_ increase.

## Methods

### Cell culture

We performed experiments on rat cultured hippocampal neurons, with approval for all animal procedures from the Yale University Animal Care and Use Committee. The methods to culture neurons are essentially the same as previously described (Brewer et al., 1993) and subsequently modified in the Boron laboratory (Chen et al., 2008). Briefly, a pregnant rat was deeply anesthetized using halothane, prior to cervical dislocation. Rat embryos (18 days) were quickly removed from the uterus and decapitated. Approximately ten brains were collected and placed in filtered HEPES-buffered solution (HBS) containing (in mM): NaCl, 143.7; KCl, 3; and HEPES, 10. Hippocampi were extracted using fine forceps and a scalpel and then exposed to 0.03% (w/v) trypsin (Sigma-Aldrich, St Louis, MO) dissolved in HBS for 15 min at 37°C. Using flamed Pasteur pipettes with reduced tip diameter, we triturated the tissues to disperse cells. Neurons were plated in neurobasal medium supplemented with B-27 (GIBCO-Invitrogen, Carlsbad, CA), 10% fetal calf serum (FCS), plus penicillin-streptomycin on poly-L-lysine (MP Biomedical, Irvine, CA) coated glass coverslips (Warner Instruments, Hamden CT, USA) or photoetched grid coverslips (Bellco Biotechnology, Vineland, NJ). After 3–4 h the medium was changed to a similar one without FCS. Neurons cultures were kept in a 5% CO_2_-air incubator at 37°C for at least seven and up to 61 days (average 23 days).

### Solutions

Table 1 lists the compositions of the physiological solutions, which were adjusted to 300 ± 3 mosmoles/kg using a vapor pressure osmometer (Model 5520C, Wescor, Inc., Logan UT). All solutions were delivered by syringe pumps (model #55-2222, Harvard Apparatus, South Holliston, MA, USA) at 7 mL min^–1^ through Tygon tubing that connected to a water-jacket system for warming before being delivered to a chamber (Bouyer et al., 2003). The temperature in the chamber was 37°C.

**TABLE 1:** Physiological solutions.

	1	2	3	4	5			6		
	Standard CO_2_/HCO_3_^−^ -free	Standard CO_2_/HCO_3_^−^	MAc	Ac (no CO_2_/HCO_3_^−^)	pAc (OOE)			pMet↓ (OOE)		
Components					A	B	Mixture	A	B	Mixture
NaCl	128	102	110	128	97	105	101	113	105	109
NaH_2_PO_4_	1.3	1.3	1.3	1.3	2.6		1.3		2.6	1.3
NaHCO_3_	0	22	14	0	44		22	28		14
KCl	3	3	3	3	6		3	6		3
MgCl_2_	2	2	2	2		4	2	4		2
CaCl_2_	2	2	2	2		4	2	4		2
Glucose	10	10	10	10		20	10		20	10
CO_2_ (%)	0	5	5	0	10	0	5	10	0	5
HEPES	32.5	32.5	32.5	32.5		65	32.5		65	32.5
pH					7.40	7.18		7.20	7.43	
Final pH	7.40	7.40	7.20	7.20			pH 7.20			pH 7.40

The concentrations are in mM, except for CO_2_ given in %. All solutions were titrated to the indicated pH at 37°C and osmolarities were adjusted to 300 ± 003 mOsm. The two out-of-equilibrium (OOE) were generated by rapid mixing as previously described (Zhao et al., 1995; 2003). Note that all nominally CO_2_-free solutions were vigorously gassed with 100% O_2_.

To generate CO_2_/HCO^−^_3_ out-of-equilibrium solutions, we followed the procedures originally described by the Boron laboratory (Zhao et al., 1995; 2003; Zhou et al., 2005), and we adapted them for hippocampal neuron solutions. For a review, see (Boron, 2004). In addition, the accompanying *Hypothesis and Theory* contribution contains a figure and an examples of how we implemented the OOE approach in the present study (Bouyer et al., 2024). Briefly the contents of two syringes (having the composition A and B in Table 1) were rapidly mixed at T connector connected to short Tygon tubing containing nylon mesh to promote mixing. The short length of Tygon tubing filled with mesh was connected to a chamber with a channel 2.5 mm wide × 14 mm long to promote laminar flow.

### Fluorescence measurements

We followed the same protocol previously described for fluorescence measurements in cultured neurons (Bouyer et al., 2004). Neurons were loaded with the pH-sensitive dye 2′,7′-bis-2-carboxyethyl)-5(and-6) carboxyfluorescein (BCECF) by incubating cells at room temperature for ∼15 min in solution 1 (Table 1) containing 10 μM of the esterified BCECF (Molecular Probes Inc., Eugene, OR). Fluorescence measurements started ∼5 min after we began to flow solution one or 2 (Table 1) through the chamber to warm cells and flush unhydrolyzed BCECF.

We used an Olympus IX70 inverted microscope equipped for epi-fluorescence (oil immersion ×40 objective, NA 1.35, with a ×1.5 magnification knob) to locate neurons on the field. The light was generated by 75-W xenon arc lamp and the two excitations wavelength of 440 nm and 490 nm were obtained by two excitations filters (440 ± 5 nm and 495 ± 5 nm, Omega Optical Inc., Brattleboro, VT) mounted on a filter wheel (Ludl Electronic Products Ltd., Hawthorne, NY) in the excitation light path. Selected neutral density filters (Omega Optical, Inc.), mounted on a second wheel, were used to equalize as nearly as possible the emitted light and avoid over-illumination of the cells. The excitation light was directed to the cells via a long-pass dichroic mirror (DM 510, Omega Optical Inc.), and we collected the emission light via a band-pass filter (530 ± 35 nm, Omega Optical Inc.) connected to intensified CCD camera (Model 350F, Video Scope International LTD, Dulles, VA). We averaged signals from 4 video frames at an acquisition rate of ranging from once every 2.5–20 s; a shutter on the filter wheel protected the cells and filters from the light between acquisitions. The data acquisition was controlled by software developed in our laboratory using the Optimas (Media Cybernetics, Inc., Silver Spring, MD) platform. Using Optimas, we delineated an area of interest (AOI) corresponded mostly to the soma of the cell and the pixel intensity of the AOI at 490 (I_490_) was divided by the pixel intensity at 440 (I_440_) nm. The fluorescence ratio I_490_/I_440_ was converted to pH_i_ values by using the high-K^+^/nigericin technique (Thomas et al., 1979), as modified in the Boron laboratory to obtain a one-point calibration at pH_i_ 7.00 (Boyarsky et al., 1988).

We generated, in a separate set of experiments, the calibration curve for BCECF, by flowing through the chamber 10 different solutions with pH values ranging from 5.8 to 8.5 to generate the parameters for the aforementioned one-point calibration. The best fit value pK was 7.13 ± 0.005 (SD), and the *b* value (i.e., the difference between R_max_ and R_min_) was 1.97 ± 0.009 (SD). High-K^+^/nigericin solutions were delivered to the chamber though a system independent of that used to superfuse the cells to avoid nigericin contamination in the lines during the actual experiments (Richmond and Vaughan-Jones, 1997; Bevensee et al., 1999). At the end of each experiment, we washed extensively the chamber with 70% ethanol and deionized water.

### Definitions

#### State

Our definitions of the states of MAc “resistance” vs MAc “sensitivity” follow the criteria adopted by Salameh et al. (2014). State applies to the size of a single pH_i_ change (ΔpH_i_) during a single acid-base challenge. In a twin-pulse protocol, each challenge has its own state. For each challenge, a neuron is resistant when (∆pH_i_/∆pH_o_) ≤ 40%, where ΔpH_i_ is the change in pH_i_ caused by ΔpH_o_, the imposed change in pH_o_. Conversely a neuron is sensitive when (∆pH_i_/∆pH_o_) is larger than 40%. Although figure 40% is somewhat arbitrary, it coincides with a natural break in the data of Salameh et al. (2014). In the twin-pulse protocols in Figure 3B through Figure 9B in Results, we use the subscript “1” to refer to the first of the two challenges, and the subscript “2” to refer to the second. In these seven figures, we plot (ΔpH_i_)_1_ for each neuron on the *x*-axis, and the corresponding (ΔpH_i_)_2_ on the *y*-axis. We indicate the resistant-sensitive boundaries by dashed blue lines, vertical for (ΔpH_i_)_1_ and horizontal for (ΔpH_i_)_2_.

“State” is defined by the location of the cell on the [(ΔpH_i_)_1_,(ΔpH_i_)_2_] coordinate system, with respect to the dashed blue lines. Thus, if a neuron is to the left of the vertical dashed blue line, the state is MAc_1_ sensitive; if below the horizontal dashed blue line, the state is MAc_2_ sensitive. If the neuron lies on the opposite side of either line, the state is resistant. The intersecting dashed blue lines define four “quadrants” that we will discuss, with examples, in conjunction with Figure 3B.

#### Behavior

Our definitions of the behaviors of “adaptation” vs “consistency” vs “decompensation” also follow the criteria adopted by Salameh et al. (2014). “Behavior” has meaning only during a twin-pulse protocol in which a cell (1) is subjected to acid-base challenge #1, (2) is allowed to recover, and (3) is subjected to acid-base challenge #2. The pH_i_ changes during the two challenges are (ΔpH_i_)_1_ and (ΔpH_i_)_2_. The determination of behavior revolves around the hourglass analysis (see B panels in Figure 3 through Figure 9) In brief, the dashed gray line that slopes upward from lower left to upper right at 45° represents identity, that is, (ΔpH_i_)_1_ = (ΔpH_i_)_2_. The sets of solid gray curves shaped like a tilted hour glass indicate our upper and lower confidence limits, based on (1) a requirement that (ΔpH_i_)_2_ be (10^0.05^–1), which is ∼12.2%, greater than (ΔpH_i_)_1_ in the case of the upper asymptote of the hourglass or (10^0.05^–1) less than (ΔpH_i_)_1_ in the case of the lower asymptote, and (2) an assumed experimental uncertainty of ±0.02 pH units. Thus,

$$Upper\;asymptote:{\left(\Delta {\text{pH}}_{i}\right)}_{2}={\left(\Delta {\text{pH}}_{i}\right)}_{1}+\left(\left[10^{\left(+0.05\right)}\right]-1\right)\cdot \left|{\left(\Delta {\text{pH}}_{i}\right)}_{1}\right|+0.02$$ (1)

$$Lower\;asymptote:{\left(\Delta {\text{pH}}_{i}\right)}_{2}={\left(\Delta {\text{pH}}_{i}\right)}_{1}\;-\;\left(\left[10^{\left(+0.05\right)}\right]-1\right)\cdot \left|{\left(\Delta {\text{pH}}_{i}\right)}_{1}\right|\;-0.02$$ (2)

where |(ΔpH_i_)_1_| indicates the absolute value.

“Behavior” is defined by the location of the cell on the [(ΔpH_i_)_1_, (ΔpH_i_)_2_] coordinate system, with respect to the upper and lower asymptotes. Thus, if (∆pH_i_)_2_ lies above the upper asymptote of the hourglass—that is, when (ΔpH_i_)_2_ is sufficiently > (ΔpH_i_)_1_—Salameh et al. (2014) defines the neuron as showing adaptation. If (ΔpH_i_)_2_ lies within the limits of the hourglass)—that is, (ΔpH_i_)_2_ ≅ (ΔpH_i_)_1_—Salameh et al. defines the neuron as showing consistency. Finally, if (ΔpH_i_)_2_ lies below the lower asymptote of the hourglass—that is, when (ΔpH_i_)_2_ is sufficiently < (ΔpH_i_)_1_—Salameh et al. defines the neuron as showing decompensation. We will provide examples in conjunction with Figure 3B.

### Data analysis and statistics

For each pH_i_ experiment, we computed the fractional rate of the BCECF dye loss (*–k*_*440*_) to assess cell viability with time (Bevensee et al., 1995). We choose to reject experiments with a rate*–k*_*440*_ > 5%.mn^−1^. Data are reported as mean ± SD, followed by the number of cells (n), the number of coverslips (N), and the number of cultures ( N ). Data were obtained from at least three different batches (i.e., obtained from at least three different litters of pups) of cultured cells. The SD values were computed on the basis of n. Means were compared using, as indicated, paired and unpaired Student’s *t-tests* (two tails), using Microsoft Excel Analysis ToolPak or Kaleidagraph (Synergy Software, Perkiomen PA, USA). Proportions were compared using z-tests; *p* < 0.05 was considered significant. Curve fitting was performed using Kaleidagraph. Correlation strength for linear fits was assessed by *R* ^*2*^ values, and we considered a correlation to be weak when *R* ^*2*^ < 0.2; mild, for 0.2–0.4; moderate, 0.4–0.6; moderately strong for 0.6–0.8; and “strong linear relationship” for 08–1.0, as previously described (Vittinghoff et al., 2005; cited in Salameh et al., 2014). Linear mixed-effects models were used to incorporate the culture date as a random effect, with likelihood ratio tests used to ascertain the significance of the culture date, using the lmerTest package in R (R version 4.4.1, https://www.r-project.org/).

## Results

### Ability of metabolic acidosis to change pH_i_

In a previous study (Bouyer et al., 2004), we found that exposing cultured rat hippocampal neurons (a total of 14) to a single challenge of metabolic acidosis caused relatively little fall in pH_i_ in the majority of HC neurons, and a larger acidification in a minority of HC neurons. In the present study, we subject a much larger number of HC neurons to extracellular MAc (5% CO_2_/14 mM HCO_3_^−^, pH = 7.20; solution 3, Table 1). Moreover, we now impose two sequential acid-base challenges per cell, and exploit out-of-equilibrium solutions to tease apart the contributions of a decreased pH_o_ *per se* and a decreased [HCO_3_^−^]_o_ *per se*. For HC neurons in which we present MAc as the first acid-base challenge, we find that the average MAc-induced pH_i_ change (measured at times that we judged pH_i_ to be approximately stable) is −0.11 ± 0.10 (n = 235 cells, N = 86 coverslips, N = 35 cultures), where the negative sign denotes a pH_i_ decrease.

**FIGURE 1:**
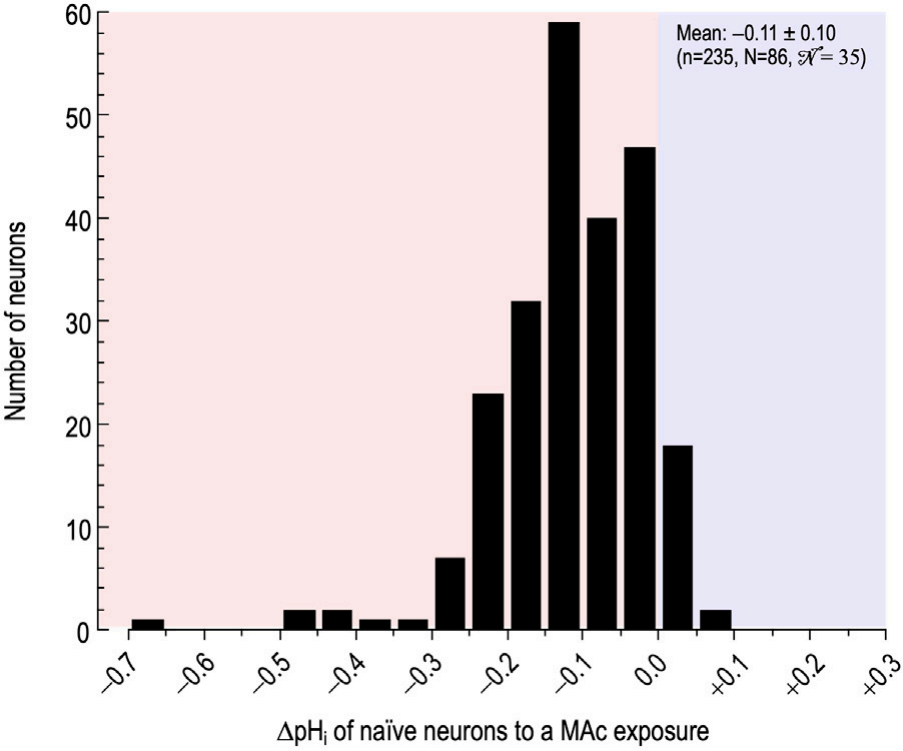
Effect of metabolic acidosis (MAc) on the pH_i_ of naïve rat hippocampal neurons. The histogram represents the distribution of the change in pHi (∆pH_i_)—judged after a time in MAc when we judged pH_i_ to be approximately stable—of neurons, caused by switching the extracellular solution from standard CO_2_/HCO_3_^−^ (solution 2, Table 1) to MAc (solution 3, Table 1). Here, we restrict the analysis to naïve neurons, that is, those for which MAc was the first challenge. The bin width is 0.05 pH units. n, number of neurons; N number of cover slips; N , number of cultures. The MAc-induced ΔpH_i_ was 0.11 ± 0.10 (mean ± SD).

Figure 1 summarizes the distribution of pH_i_ changes (∆pH_i_). Using the definitions of MAc-resistant and MAc-sensitive cells (see Definitions^1^) and previously established (Salameh et al., 2014) we find that 90 neurons (∼38%) were MAc-resistant and 145, MAc-sensitive (∼62%). These percentages are nearly identical to those from a study on cultured mouse HC neurons (Salameh et al., 2014). We will refer to MAc-resistant vs MAc-sensitive as physiological “states.”

Note that, although the vast majority of neurons respond to MAc with the expected acidification (i.e., negative ΔpH_i_ values in Figure 1, reddish background), in few cells, MAc paradoxically elicits an alkalinization (i.e., positive ΔpH_i_ values at the extreme right of Figure 1, blueish background).

### Relationships between MAc-induced ΔpH_i_ and time in culture, and initial steady state pH_i_

#### Time in culture and ΔpH_i_ during MAc

**FIGURE 2:**
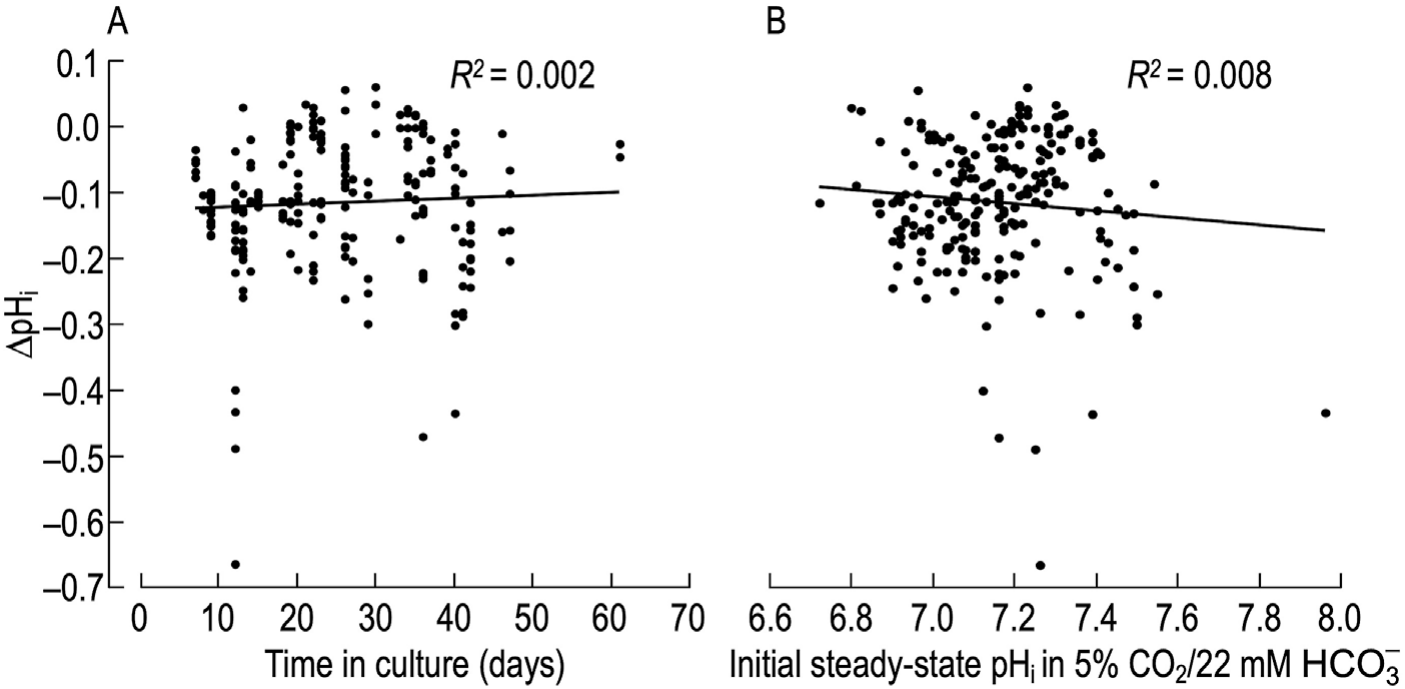
Relationship between pH_i_ change induced by metabolic acidosis (MAc) in naïve rat hippocampal neurons *versus* time in culture and initial steady-state pHi **(A)**, change in pH_i_ (ΔpH_i_)—judged after a time in MAc when we judged pH_i_ to be approximately stable—vs. time in culture. We induced the ΔpH_i_ by switching the extracellular solution from standard CO_2_/HCO_3_^−^ (solution 2, Table 1) to MAc (solution 3, Table 1). As in Figure 1, we restrict the analysis to naïve neurons, that is, those for which MAc was the first challenge. The linear fit regression fit shows no correlation between ΔpH_i_ vs time in culture. **(B)**, change in pH_i_ vs initial steady-state pH_i_ value. These are the same ΔpH_i_ data as in panel **(A)**. The linear regression fit shows no correlation between ΔpH_i_ and pH_i_ values.

To determine whether the pH_i_ response to MAc depends on time in culture, in Figure 2A we plot the ∆pH_i_ of naïve neurons as a function of time, and find no correlation (*R* ^*2*^ = 0.002).

#### Impact of culture date on ΔpH_i_

We maintained uniform conditions for each of the seven twin-challenge protocols that we present in the remainder of Results: MAc-MAc, Ac-MAc, MAc-Ac, pAc-MAc, MAc-pAC, pMet↓-MAc and MAc-pMet↓. Thus, we should not expect to observe systematic differences in ∆pH_i_ according to the culture date. To examine that possibility, we used a linear mixed-effects model to determine if culture date represents a significant predicator of the relationship between the pH_i_ just before the first of two challenges and the ΔpH_i_ during that challenge. Of the seven protocols, the random effect of culture date was significant only for two, MAc-MAc (χ^2^(1) = 15.8, *p* < 0.001) and pAc-MAc (χ^2^(1) = 28.2, *p* < 0.001). The most likely explanation is that these two instances of significance reflect random fluctuations, rather than biological effects, inasmuch as experimental conditions were uniform for each of the seven protocols.

#### Correlation between initial pH_i_ and ΔpH_i_

We know from work of the Boron and the Church laboratories on HC neurons that, upon switching from a HEPES-buffered to a CO_2_/HCO_3_^−^-buffered extracellular solution, the initial steady-state pH_i_ impacts the direction (i.e., acidification vs alkalinization) and magnitude of the ensuing pH_i_ response (Schwiening and Boron, 1994; Bevensee et al., 1996; Smith et al., 1998). To test the hypothesis that the initial steady-state pH_i_ in CO_2_/HCO_3_^−^ may also influence the degree of MAc-induced acidification, in Figure 2B we plot the ΔpH_i_ of naïve neurons vs the initial pH_i_, and find no correlation between the two parameters (*R* ^*2*^ = 0.008). Therefore, we conclude that factors other than time in culture and initial steady-state pH_i_ are responsible for the different pH_i_ responses observed during an exposure to MAc.

We reach a similar conclusion when examining all initial-pH_i_/∆pH_i_ data pairs in the four protocols in which MAc is the first challenge—that is, MAc_1_—experienced by naïve neurons: MAc-MAc, MAc-Ac, MAc-pAC and MAc-pMet↓. The best fit regression line for these data (not shown) has *R* ^*2*^ = 0.00056, indicating no relationship.

#### Relationship between (pH_i_)_1_ and (pH_i_)_2_

In six of the seven twin-pulse protocols—all except Ac-MAc—we found a positive and significant correlation (*p* < 0.001; not shown) between the pH_i_ before the first challenge, (pH_i_)_1_, and the pH_i_ before the second challenge, (pH_i_)_2_. That is, higher (pH_i_)_1_ values correlate with higher (pH_i_)_2_ values.

#### Twin MAc exposures

In work on cultured mouse HC neurons, Salameh et al. (2014) monitored pH_i_ while subjecting the cells to two consecutive MAc challenges—MAc_1_ and MAc_2_—separated by a period of recovery. As set out in Methods (see Definitions), Salameh et al. (2014) characterized cell “state” as resistant vs sensitive based upon the magnitude of (ΔpH_i_)_1_ during MAc_1_ and (ΔpH_i_)_2_ during MAc_2_. Comparing (ΔpH_i_)_2_ vs the previous (ΔpH_i_)_1,_ those authors also defined neuron “behavior” as “adaptation”, “consistency,” and “decompensation” based on the position of the neuron on a [(ΔpH_i_)_1_, (ΔpH_i_)_2_] coordinate system, with respect to the hourglass defined by Equations 1, 2 in Methods.

Salameh et al. worked with mouse (rather than rat) HC neurons, and cultured the HC neurons together with abundant (rather than depleted) astrocytes. In this first Results section, we ask whether the Salameh results for twin MAc pulses are generalizable to the present conditions for rat HC neurons.

##### Sample pHi records

**FIGURE 3:**
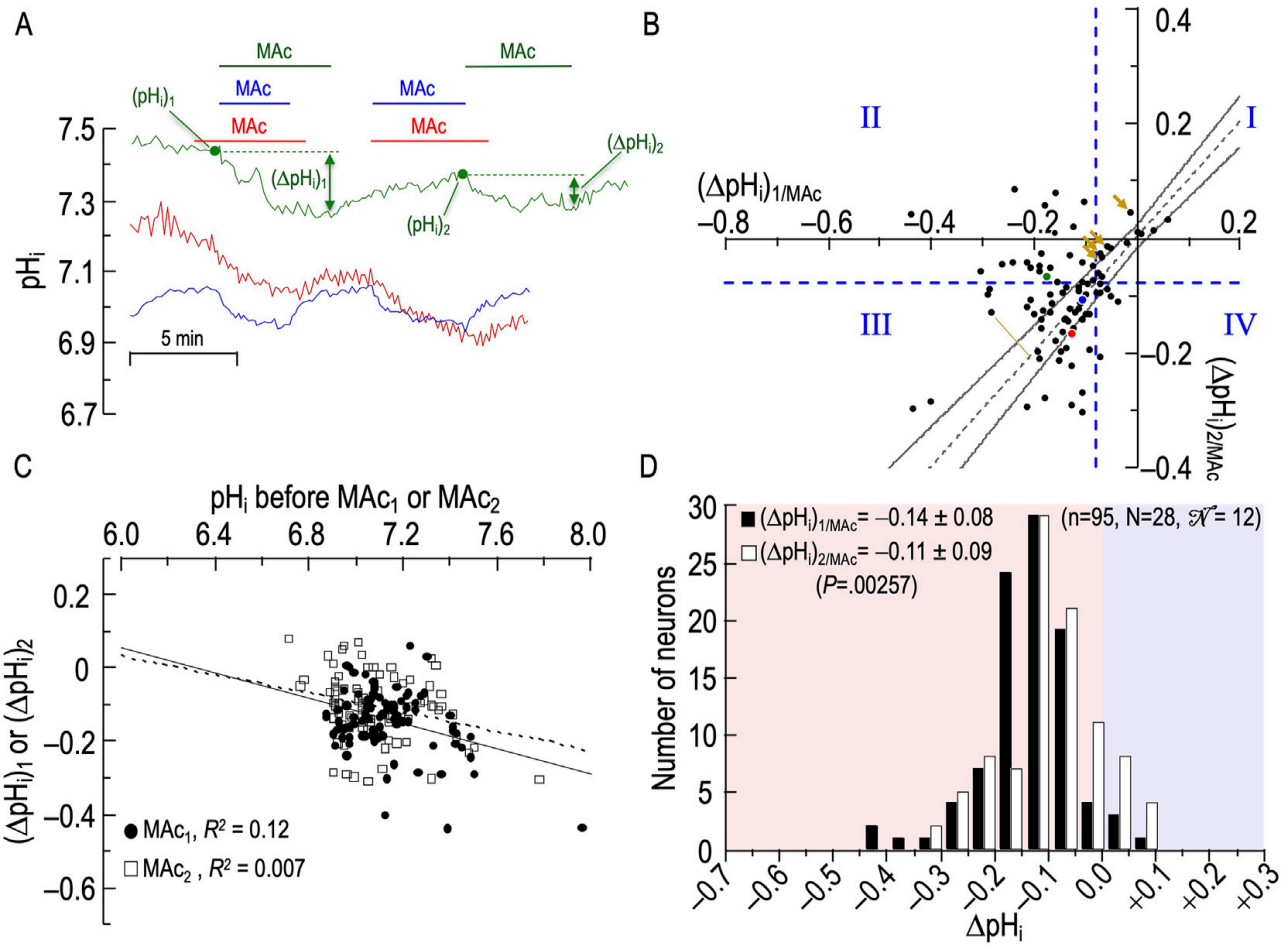
Effect of twin exposures to metabolic acidosis (MAc) on pH_i_ of rat hippocampal neurons **(A)**, examples of pH_i_ responses of three neurons to twin challenges in which we switched from standard CO_2_/HCO_3_^−^ (solution 2, Table 1) to MAc (solution 3, Table 1). Unless otherwise indicated, the extracellular solution was standard CO_2_/HCO_3_^−^ (solution 2, Table 1). **(B)**, relationship between the ΔpH_i_ during the first exposure to MAc—that is, (ΔpH_i_)_1/MAc_—and ΔpH_i_ during the second exposure to MAc—that is, (ΔpH_i_)_2/MAc_. The vertical dashed blue line is the resistant-sensitive demarcation for MAc_1_, and the horizontal dashed blue line is the same for MAc_2_. Both demarcations are at ΔpH_i_/ΔpH_o_ = 40%. Together, these dashed lines define quadrants Q_I_ through Q_IV_ and “pH_i_ states.” Because the blue and red dots, representing the blue and red neurons in **(A)**, are in Q_III_, those neurons are both MAc_1_ sensitive and MAc_2_ sensitive. Because the green dot is in Q_II_, the green neuron in panel A is MAc_1_ sensitive but MAc_2_ resistant. The four bronze arrows indicate four neurons that were MAc resistant in both challenges. The dashed gray line represents the line of identity, for which (ΔpH_i_)_1/MAc_ = (ΔpH_i_)_2/MAc_. The upper and lower bending asymptotes—the hourglass, which defines “pH_i_ behavior”—represent the confidence interval as described in Methods. Using these criteria, the blue neuron in panel A displays consistency because the blue point in **(B)** lies within the hourglass, that is, (ΔpH_i_)_2_ ≅ (ΔpH_i_)_1_. The green neuron displays adaptation because the green point lies above the upper asymptote of the hourglass. The red neuron displays decompensation because the red point lies just below the lower asymptote of the hourglass. **(C)**, dependence of (ΔpH_i_)_1_ (black circles) or (ΔpH_i_)_2_ (white squares) on the initial pH_i_ before MAc_1_ and MAc_2_, respectively. The solid and dashed lines represent the linear regressions for MAc_1_ and MAc_2_, respectively. **(D)**, frequency distribution of (ΔpH_i_)_1/MAc_ (black bars) and (ΔpH_i_)_2/MAc_ (white bars) with a pH_i_ bin width of 0.05 pH units. In the upper left, we report means ± standard deviation as well as the *p*-value (paired t-test, two-tails). In the upper right, we report n, number of neurons; N, number of cover slips; N , number of animals/cultures.

Figure 3A shows pH_i_ records—colored blue, green, and red—from three rat HC neurons, each challenged with two consecutive MAc pulses. For the green record, we label [1] (pH_i_)_1_, the pH_i_ just before MAc_1_; [2] (ΔpH_i_)_1_, the pH_i_ change by the end of MAc_1_; [3] (pH_i_)_2_, the pH_i_ just before MAc_2_; and [4] (ΔpH_i_)_2_, the pH_i_ change by the end of MAc_2_. The blue and red^2^ pH_i_ records show neurons with relatively large acidifications during the first MAc exposure (i.e., they are MAc_1_ sensitive), and relatively large acidifications during the second MAc exposure (i.e., they are MAc_2_ sensitive as well). The green pH_i_ trace shows a neuron with a relatively large acidification during the first MAc exposure (i.e., it is MAc1 sensitive), but a smaller pH_i_ change during the second MAc exposure (i.e., it is MAc2 resistant).

Figure 3B is the basis for three analyses, identified below as #1 (State), #2 (Behavior), and #3 (*d*_±_). The first two are based on approaches of Salameh et al. (2014) as defined in Methods. The third is novel to the present paper Figure 3A shows how 95 individual neurons (n = 95, N = 28, N = 12) respond to MAc_1_ and MAc_2_. The *x*-axis represents (ΔpH_i_)_1_ and the *y*-axis, (ΔpH_i_)_2_. Each of the 95 points represents a separate neuron, with the three colored points representing the three neurons in Figure 3A. Table 2 summarizes some of the statistics from the protocols of Figure 3 through Figure 9.

**TABLE 2:** Summary of data sets represented in Figure 3 through Figure 9

Protocol	Figure	Table	n	N	N	*d*_Absolute_	*d*_±_	Corrected *d*_±_	Δ(pH_i_)_1_	Δ(pH_i_)_2_
MAc-MAc	3	3	95	28	12	0.055	+0.024 ± 0.075 (*p* = .00258)		−0.14 ± 0.08	−0.11 ± 0.09
Ac-MAc	4	4	39	17	10	0.076	+0.015 ± 0.121 (*p* = .434)		−0.11 ± 0.12	−0.09 ± 0.09
MAc-Ac	5	5	37	13	7	0.107	+0.023 ± 0.138 (*p* = .309)		−0.11 ± 0.09	−0.07 ± 0.13
pAc-MAc	6	6	47	13	7	0.058	−0.015 ± 0.083 (*p* = .211)		−0.10 ± 0.10	−0.12 ± 0.10
MAc-pAc	7	7	37	19	6	0.044	+0.020 ± 0.054 (*p* = .0275)		−0.10 ± 0.10	−0.07 ± 0.06
pMet↓-MAc	8	8	52	17	12	0.078	−0.047 ± 0.082 (*p* = .000110)	−0.022	−0.04 ± 0.10	−0.11 ± 0.07
MAc-pMet↓	9	9	61	24	9	0.094	+0.094 ± 0.069 (*p* = 2.28×10^−15^)		−0.07 ± 0.08	−0.06 ± 0.06

^a^ Column headings: Figure, Figure number for data set; Table, Table number for data set; n, number of neurons; N, number of cover slips; N , number of culture preparations; *d*_Absolute_, average absolute (positive) distance from point to line of identity; *d*_±_, mean signed distance from point to line of identity; Corrected *d*_±_, *d*_±_ corrected for shift between (ΔpH_i_)_1/pMet↓_ vs (ΔpH_i_)_1/MAc_, in Figure 8 vs Figure 9; (ΔpH_i_)_1_, pH_i_ change during first acid-base challenge; (ΔpH_i_)_2_, pH_i_ change during second acid-base challenge. Row headings: MAc, metabolic acidosis; Ac, acidosis (in the nominal absence of CO_2_/HCO_3_^−^); pAc, pure acidosis; pMet↓, pure metabolic down (i.e., decrease in [HCO_3_^−^]_o_).

##### Analysis #1 state

As noted in Methods, we can represent state by the position of a cell with respect to the vertical dashed blue line (the resistant-sensitive demarcation for MAc_1_) and the horizontal dashed blue line (the resistant-sensitive demarcation for MAc_2_). The two dashed blue lines define four “quadrants”, Q_I_ through Q_IV_. As we will see below, the position of a neuron in one of the four quadrants describes how the state of the neuron changes^3^ (or does not change) in the progression from MAc_1_ to MAc_2_.

Consistent with our analysis of Figure 1 (a data set of 235 neurons that includes the 95 here), we see here in Figure 3B that a minority of the neurons (19/95 or 20%) lie to the right of the vertical dashed line. That is, according to the definition of Salameh et al. (2014), the state of these neurons during MAc_1_ is MAc resistant; the left side of Table 3A provides a numerical summary of these neurons. The majority (80%) of neurons, however, lie to the left of the vertical dashed line; that is, their state is sensitive during MAc_1_ (Table 3A, right). The remainder of Table 3 summarizes the state transition from MAc_1_ to MAc_2_ (Table 3B), the behavior (i.e., adaptation, consistency, decompensation) between MAc_1_ and MAc_2_ (Table 3C), and the state during MAc_2_ (Table 3D). During the second MAc challenge, 33 of 95 or 35% of the neurons lie above the horizontal dashed line in Figure 3B; that is, these neurons are resistant during MAc_2_, as also summarized in Table 3D/left. On the other hand, 65% lie below this line (Table 3D/right) and are therefore MAc_2_ sensitive. Note that the distribution of MAc-resistant to MAc-sensitive neurons is 35%/65% during MAc_2_, compared to 20%/80% during MAc_1_. That is, from MAc_1_ to MAc_2_, we see a general trend toward MAc resistance.

**TABLE 3:** State and behavior of 95 hippocampal neurons during twin pulses of metabolic acidosis (MAc).

A, state:* MAc_1_	19 neurons (20%) = resistant†		76 neurons (80%) = sensitive†		
B, State transition:† MAc_1_ → MAc_2_	12 (13%) remain Resistant [Q_I_]	7 (7%) become Sensitive [Q_IV_]	21 (22%) become Resistant [Q_II_]	55 (58%) remain Sensitive [Q_III_]	
C, Behavior:‡ MAc_1_→ MAc_2_*					Total
Adaptation	4 (4%)	0	20 (21%)	17 (18%)	41 (43%)
Consistency	8 (9%)	3 (3%)	1 (1%)	20 (21%)	32 (34%)
Decompensation	0	4 (4%)	0	18 (19%)	22 (23%)
D, State:† MAc_2_ resistant	33 (35%)		MAc2 Sensitive	62 (65%)	

^a^ State for pulse 1: resistant neurons are to the right of the vertical dashed blue line; sensitive neurons are to the left.

^b^ State for pulse 2, resistant neurons are above the horizontal dashed blue line; sensitive neurons are below.

^c^ Behaviors for pulse 1→2 transition: Adaptation (i.e., point is above the hourglass); Consistency (i.e., point is within the hourglass); Decompensation (i.e., point is below the hourglass).

**Key points:** During MAc_1_, most HC, neurons are sensitive, whereas during MAc_2_, we observe a trend toward resistance. This shift is associated with an adaptation behavior supported by an overall positive *d±* (see Table 2).

Q, quadrant; Q_I_, quadrant I (neurons that are resistant during MAc_1_, and remain resistant during MAc_2_); Q_II_, quadrant II (MAc_1_ sensitive → MAc_2_ resistant); Q_III_, quadrant III (MAc_1_ sensitive → MAc_2_ sensitive); Q_IV_, quadrant IV (MAc_1_ resistant → MAc_2_ sensitive).

Table 3B traces the fates (i.e., behavior) of the neurons identified as resistant or sensitive during MAc_1_:

• Of the 19 neurons identified as resistant during MAc_1_, 12 remain resistant during MAc_2_. By definition, these 12 MAc_1_-resistant/MAc_2_-resistant neurons all lie in the first quadrant (Q_I_, upper right) in Figure 3B.
• Of the seven previously resistant neurons become sensitive during MAc_2_ and therefore lie in Q_IV_ (lower right), which contains all MAc_1_-resistant/MAc_2_-sensitive neurons.
• Of the 76 neurons identified as sensitive during MAc_1_, 55 remain sensitive during MAc_2_, and thus lie in Q_III_, (lower left), which contains all MAc_1_-sensitive/MAc_2_-sensitive neurons.
• Finally, 21 of the 76 become resistant and thus lie in Q_II_ (upper left), which contains all MAc_1_-sensitive/MAc_2_-resistant neurons.

Thus, of the 33 neurons resistant during MAc_2_ (Table 3D), only 12 were originally resistant in MAc_1_, whereas 21 more were sensitive during MAc_1_ but became resistant. Similarly, of the 62 neurons identified as sensitive during MAc_2_ (Table 3D), 55 were sensitive during MAc_1_, and seven others that were resistant during MAc_1_ became sensitive. Thus, although shifts in state are not uncommon—the more common being from MAc_1_-sensitive to MAc_2_-resistant (i.e., Q_II_) rather than from MAc_1_-resistant to MAc_2_- sensitive (i.e., Q_IV_)—neurons tend to maintain their resistant/sensitive state-phenotypes between MAc_1_ and MAc_2_ (i.e., Q_I_ and Q_III_).

For the remainder of Results, we present tabular data analogous to that in Table 3 (see Table 4 through Table 9) but limit the presentation to a few salient features in the table legends. Thus, the above presentation of Table 3 serves as a guide for the six later tables.

**TABLE 4:** State and behavior of 39 hippocampal neurons during exposure to extracellular acidosis (Ac) and metabolic acidosis (MAc).

A, state:* Ac_1_	13 neurons (33%) = resistant		26 neurons (67%) = sensitive		
B, state transition:† Ac_1_→ MAc_2_	7 (18%) remain resistant [Q_I_]	6 (15%) become sensitive [Q_IV_]	11 (28%) become resistant [Q_II_]	15 (39%) remain sensitive [Q_III_]	
C, Behavior:‡ Ac_1_→ MAc_2_*					Total
Adaptation	2 (5%)	0	11 (28%)	2 (5%)	15 (38%)
Consistency	3 (8%)	0	0	5 (13%)	8 (21%)
Decompensation	2 (5%)	6 (15%)	0	8 (21%)	16 (41%)
D, State:† MAc_2_ Resistant	18 (46%)^Δ^		MAc_2_ Sensitive	21 (54%)^Δ^	

^a^ State for pulse 1: resistant neurons are to the right of the vertical dashed blue line; sensitive neurons are to the left.

^b^ State for pulse 2, resistant neurons are above the horizontal dashed blue line; sensitive neurons are below.

^c^ Behaviors for pulse 1→2 transition: Adaptation (i.e., point is above the hourglass); Consistency (i.e., point is within the hourglass); Decompensation (i.e., point is below the hourglass).

Q, quadrant; Q_I_, quadrant I (neurons that are “resistant” during Ac_1_, and remain resistant during MAc_2_); Q_II_, quadrant II (Ac_1_ sensitive → MAc_2_ resistant); Q_III_, quadrant III (Ac_1_ sensitive → MAc_2_ sensitive); Q_IV_, quadrant IV (Ac_1_ resistant → MAc_2_ sensitive).

^Δ^ **Key points:** In this Ac-MAc, protocol, 46% of the neurons are MAc_2_ resistant vs only 35% in the MAc-MAc, protocol (see Table 3D). A two-sample Z-test for proportions reveals that 35% MAc_2_ resistance in MAc-MAc, is not significantly different from 46% in Ac-MAc (*p* = 0.216).

##### Analysis #2: Behavior (i.e., hourglass)

The second approach for examining the data in Figure 3B is the hourglass analysis of behavior, introduced by Salameh et al. (2014) and summarized in Methods^4^, as well as the legend of Figure 3. Table 3C lists the behaviors of the 95 neurons. For example, of the 12 MAc_1_-resistant/MAc_2_-resistant neurons that lie in Q_I_, four lie above the hourglass (bronze arrows in Figure 3B); that is, their behavior is adaptation. The other eight neurons lie within the hourglass; their behavior is consistency. None of these 12 neurons lie below the hourglass; that is, none has a decompensation behavior.

In the rightmost column of Table 3C, we summarize the behaviors of all 95 neurons. For example, 41 of 95 neurons or 43%—including the one represented by the green point here and the green record in panel A—lie above the hourglass. These 41 neurons have an adaptation behavior. In fact, seven of these 41 points lie at least ∼0.02 pH units above the *x*-axis; that is, these neurons adapted to such an extent that they exhibit a frank alkalinization during MAc_2_. Table 3C also shows that 32 of 95 or 34% of the neurons—including the one represented by the blue point and record—lie within the hourglass; that is, their behavior is consistent between MAc_1_ and MAc_2_. Finally, 22 of 95 or 23% of the neurons—including the one represented by the red point and record—lie below the hourglass; that is, these neurons exhibit a decompensation behavior between MAc_1_ and MAc_2_. Note that four of these decompensating neurons were MAc_1_ resistant but MAc_2_ sensitive, whereas 18 were MAc_1_ sensitive and remained sensitive during MAc_2_ (albeit with a greater magnitude of ΔpH_i_).

##### Analysis #3: *d_±_*

The third analysis, which we introduce in the present paper is actually two closely related calculations. In the first, we compute the mean absolute distance (*d*_Absolute_) between each neuron-point to the nearest point^5^ on the line of identity (LOI). All *d*_Absolute_ values are positive. We indicate this relationship in Figure 3B with the gold line segment between one neuron in Q_III_ and the LOI. For each protocol, the mean *d*_Absolute_ value is in the seventh column of Table 2. In the second calculation, we compute the mean signed distance (*d*_±_). Individual values are positive if the corresponding point lies to the upper left of the LOI (as for the gold line segment in Figure 3B)—this is analogous to adaptation behavior, but does not take into account the confidence interval represented by the hourglass. Individual values are negative if the corresponding point lies to the lower right of the LOI analogous to decompensation behavior. Mean *d*_±_ values for each protocol are in the eighth column of Table 2. For this MAc-MAc protocol, the mean *d*_±_ is ∼ +0.024 (*p* = .00258; Table 2, row 1), which indicates that the average point is significantly to the upper-left of the LOI, consistent with an overall tendency toward an adaptation behavior.

##### ΔpH_i_ vs initial pH_i_

To determine whether the pH_i_ just before MAc_1_ or MAc_2_ correlates with the ΔpH_i_ during the subsequent MAc, in Figure 3C we plot ΔpH_i_ vs the initial pH_i_ for both MAc pulses. As described above for Figure 2B, taking all 95 points together, we find the overall correlation strength to be “absent” (see Methods^6^), both between (ΔpH_i_)_1_ and (pH_i_)_1_ (filled circles; *R* ^*2*^ = 0.12), and between (ΔpH_i_)_2_ and (pH_i_)_2_ (open squares; *R* ^*2*^ = 0.007).

Returning to Figure 3A, we see that the red record is from a neuron in which (ΔpH_i_)_1_ was relatively large, and for which the pH_i_ recovery from MAc_1_ was small, so that (pH_i_)_2_ was substantially lower than (pH_i_)_1_. Indeed, our analysis (not shown) of the 95 neurons in Figure 3 shows a weak correlation (*R* ^2^ = 0.36) between (ΔpH_i_)_1_ and the difference [(pH_i_)_2_ – (pH_i_)_1_]. That is, as the acidification during MAc_1_ increases in magnitude, (pH_i_)_2_ tends to fall increasingly below (pH_i_)_1_.

##### Frequency distributions

Figure 3D shows the frequency distributions of ΔpH_i_ for both MAc_1_ and MAc_2_ and reveals a statistically significant shift to the right (i.e., smaller negative ΔpH_i_ values, larger positive ones) between MAc_1_ and MAc_2_. This result is consistent with an overall trend to adaptation and the observed positive value of *d*_±_. Note that this traditional histogram cannot capture the rich diversity among neurons, as revealed in Figures 3B, C and Table 3.

##### Summary of MAc-MAc

Most rat HC neurons have a MAc-sensitive “state”, both during MAc_1_ and MAc_2_ (Q_III_). From MAc_1_ to MAc_2_, the neurons tend to show adaptation-like “behavior”, being both above the hourglass and having a positive *d*_±_.

### Extracellular acidosis in the absence of CO_2_/HCO_3_^−^ (Ac) and then MAc

To begin exploring the role of ΔpH_o_ *per se* in producing the ΔpH_i_ observed during MAc, we examine neurons exposed to a CO_2_/HCO_3_^−^-free solution at pH 7.40 (solution 1, Table 1) and then subject the neurons—in the continuing absence of CO_2_/HCO_3_^−^—to extracellular acidosis (Ac; pH_o_ 7.20; solution 4, Table 1). To compare this Ac-induced pH_i_ response with our previous MAc data, we return the neurons to the pH-7.4 solution 1, then switch to our standard CO_2_/HCO_3_^−^ solution (solution 2, Table 1), and then finally impose a standard MAc (solution 3, Table 1).

#### Nomenclature

We do not expect the response to Ac—or other single-parameter challenges presented later—to be the same as the response to MAc. Ac, for one, occurs in the nominal absence of CO_2_ and HCO_3_^−^, where minimal CO_2_ or HCO_3_^−^ is present for sensing or effector mechanisms (e.g., transport of HCO_3_^−^-related species). Moreover, we do not expect the response to the twin challenge Ac-MAc—and other twin challenges presented later—to be the same as the response to MAc-MAc. Nevertheless, for the sake of consistent comparisons, we analyze them all as we do for MAc-MAc. Thus, we will use terms like “resistant” and “adaptation” the same way as we use them for MAc and MAc-MAc, respectively, understanding that this use is for our convenience and does not necessarily imply equivalence of the physiological challenges.

#### Sample pH_i_ records

**FIGURE 4:**
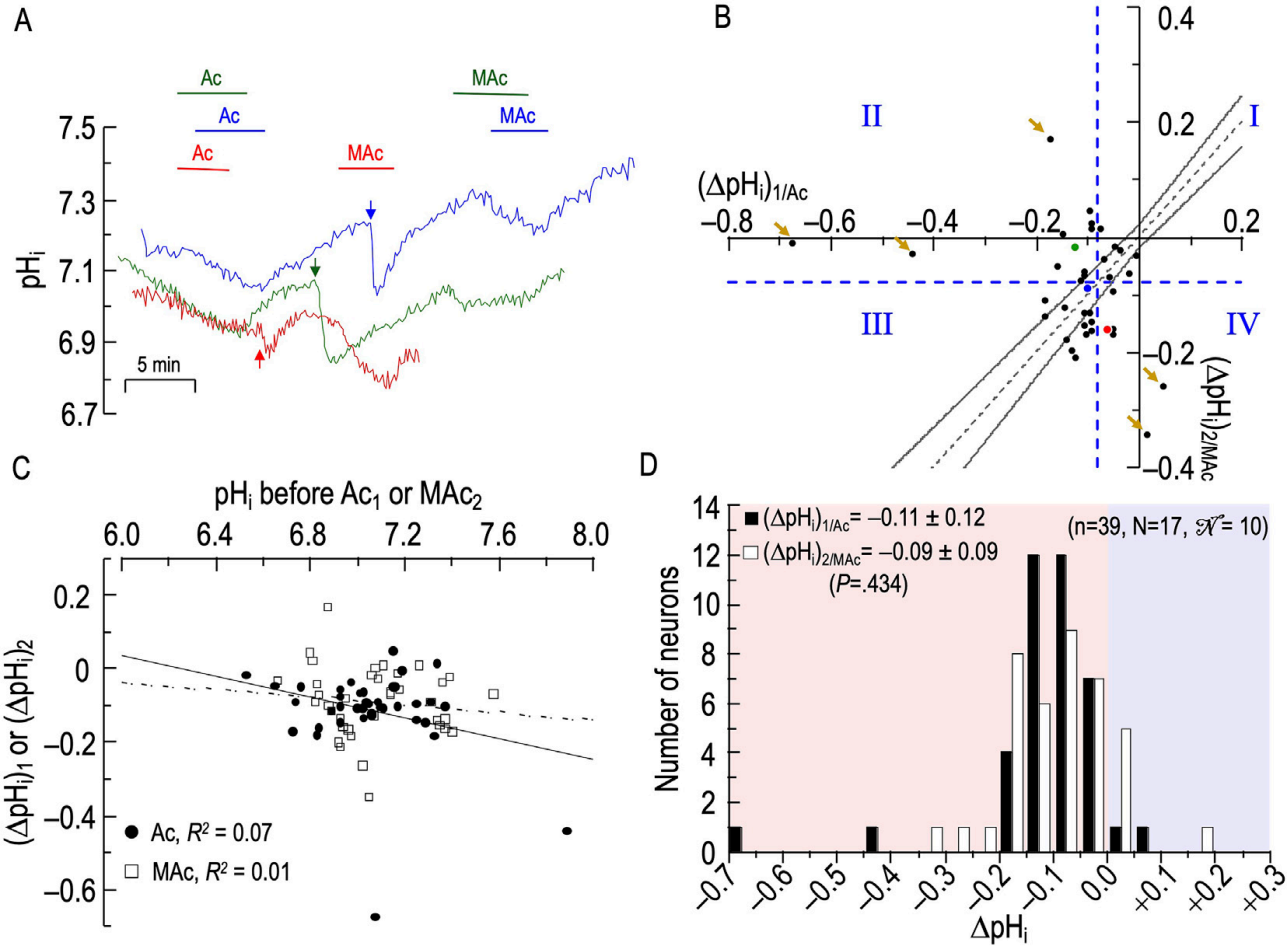
Effect extracellular acidosis (Ac) followed by metabolic acidosis (MAc) on the pH_i_ of rat hippocampal neurons **(A)**, examples of the pH_i_ responses in three hippocampal neurons to an exposure to Ac (solution 4, Table 1) and then to MAc (solution 3, Table 1). Before the arrows, unless otherwise indicated, the bath solution was our standard nominally CO_2_/HCO_3_^−^-free solution (solution 1, Table 1). After the arrows, unless otherwise indicated, the bath solution was standard CO_2_/HCO_3_^−^ (solution 2, Table 1). **(B)**, relationship between the ΔpH_i_ during exposure to Ac—that is, (ΔpH_i_)_1/Ac_—and ΔpH_i_ during exposure to MAc—that is, (ΔpH_i_)_2/MAc_. The horizontal and vertical dashed blue lines are the resistant-sensitive demarcations that define quadrants I–IV and “pH_i_ states” (see Figure 3). The blue dot that represents the blue neuron in panel A is in Q_III_ (i.e., neuron is both Ac_1_ sensitive and MAc_2_ sensitive). The green dot is in Q_II_ (i.e., green neuron in panel A is Ac_1_ sensitive but MAc_2_ resistant). The red dot is in Q_IV_ (i.e., red neuron in panel A is Ac_1_ resistant but MAc_2_ sensitive). The five bronze arrows indicate neurons that are particularly distant from the hourglass. Moreover, all five are either near or paradoxically above the *x*-axis, or to the right of the *y*-axis. The dashed gray line is the line of identity; the gray hourglass represents the confidence interval and defines “pH_i_ behavior” (see Figure 3). Because the blue point lies within the hourglass, the behavior of the blue neuron is consistency. The green point lies above the upper asymptote of the hourglass; the behavior is adaptation. The red neuron lies below the lower asymptote of the hourglass; the behavior is decompensation. **(C)**, dependence of (ΔpH_i_)_1_ (black circles) on the initial pH_i_ before Ac_1_, which we refer to as (ΔpH_i_)_1/Ac_, or dependence of (ΔpH_i_)_2_ (white squares) on the initial pH_i_ before and MAc_2_, which we refer to as (ΔpH_i_)_2/MAc_. The solid and dashed lines represent the linear regressions for Ac_1_ and MAc_2_, respectively. **(D)**, frequency distribution of (ΔpHi)_1/Ac_ (black bars) and (ΔpHi)_2/MAc_ (white bars) with a pH_i_ bin width of 0.05 pH units. In the upper left, we report means ± standard deviation as well as the *p*-value (paired t-test, two-tails). n, number of neurons; N, number of cover slips; N , number of animals/cultures.

Figure 4A shows the pH_i_ responses of three neurons to the aforementioned protocol. We focus first on the blue record. Imposing Ac_1_ (first challenge) produces a slow decrease in pH_i_, the final magnitude of which is (ΔpH_i_)_1/Ac_. Returning pH_o_ to 7.40 produces a slow pH_i_ recovery, presumably due to Na-H exchange (Baxter and Church, 1996; Bevensee et al., 1996). Adding CO_2_/HCO_3_^−^ (downward blue arrow) produces a rapid pH_i_ decrease due to CO_2_ influx, followed by a brisk pH_i_ recovery due to acid extrusion mediated to some extent by Na-H exchangers, but predominantly by Na^+^-coupled HCO_3_^−^ transporters (Schwiening and Boron, 1994; Baxter and Church, 1996; Bevensee et al., 1996). Here, the evidence that HCO_3_^−^ transport is dominant is the far more rapid pH_i_-recovery in the presence of CO_2_/HCO_3_^−^, even though total intracellular buffering power must have been far higher in CO_2_/HCO_3_^−^ (Roos and Boron, 1981; Boron, 2004). The subsequent MAc_2_, like the preceding Ac_1_, produces a slow pH_i_ decline; the pH_i_ change—(ΔpH_i_)_2/MAc_—is approximately the same magnitude as (ΔpH_i_)_1/Ac_.

The green and red traces in Figure 4A represent two other neurons. Note that, whereas the pH_i_ response to MAc generally results in a clear shift in steady-state pH_i_ (see Figure 3A), the response to Ac often results in a continuous downward drift in pH_i_. Thus, our (ΔpH_i_)_1/Ac_ values do not necessarily reflect a shift in steady-state pH_i_ *per se*, but an evolving pH_i_ change over a time period similar to that in MAc challenges. We present these Ac_1_ data so that the reader may be aware that pH_o_ changes under non-physiological conditions often lead to unexpected consequences. The pH_i_ data for MAc_2_ are rather nominal except for the red trace, which may have been drifting downward just before the MAc_2_ challenge.

#### Analyses #1–3

Figure 4B and Table 4 summarize state and behavior for the full Ac-MAc dataset (n = 39, N = 17, N = 10).^7^ Figure 4B reveals that most neurons have relatively low absolute values of (ΔpH_i_)_1/Ac_ and (ΔpH_i_)_2/MAc_, and most tend to cluster near the hourglass. The notable exceptions are five neurons (bronze arrows) that are rather distant from the hourglass, three neurons in Q_II_, and two others in Q_IV_.

The blue point, which corresponds to the neuron represented by the same color in Figure 4A, lies in Q_III_ (i.e., its “state” fulfills the MAc criteria for Ac_1_ sensitivity and is MAc_2_ sensitive) and falls within the hourglass, near the LOI (its “behavior” fulfills the MAc-MAc criteria for consistency (see Figures 3A,B).

The green point lies in Q_II_ (i.e., its states are Ac_1_ sensitive but MAc_2_ resistant), and above the hourglass (i.e., behavior is adaptation).

The red point lies in Q_IV_ (i.e., states are Ac_1_ resistant and MAc_2_ sensitive), and just outside of the lower bound of the hourglass (i.e., behavior is decompensation).

The *d*_±_ of +0.015 (Table 2, row 2) is not significantly different from zero, consistent with our subjective impression of a general clustering near the hourglass.

#### ΔpH_i_ vs initial pH_i_

Figure 4C shows that the correlation strengths, taking all 39 points together, were “absent” for both (ΔpH_i_)_1/Ac_ vs (pH_i_)_1_, and for (ΔpH_i_)_2/MAc_ vs (pH_i_)_2_.

#### Frequency distributions

Figure 4D shows the frequency distributions of ΔpH_i_ for both Ac_1_ and MAc_2_. Unlike the situation for MAc-MAc (see Figure 3D), (ΔpH_i_)_2_ is not significantly different from (ΔpH_i_)_1_ in the Ac-MAc protocol. Note that the MAc response during pulse two in the Ac-MAc protocol (ΔpH_i_)_2/MAc_ = −0.09 ± 0.09 (see Figure 4D) is not significantly different from its counterpart in the MAc-MAc protocol ((ΔpH_i_)_2/MAc_ = −0.11 ± 0.09, *p* = 0.333, unpaired t-test, in Figure 3D).

#### Summary of Ac-MAc

Ac_1_ often causes a slow, continuing decline in pH_i_, and—compared to MAc-MAc—has a negligible effect on (ΔpH_i_)_2/MAc_.

### MAc and then Ac

In this new experimental series, we reverse the order of the challenges in Figure 4, exposing the neurons first to MAc then to Ac (in absence of CO_2_/HCO_3_^−^).

#### Sample pH_i_ records

**FIGURE 5:**
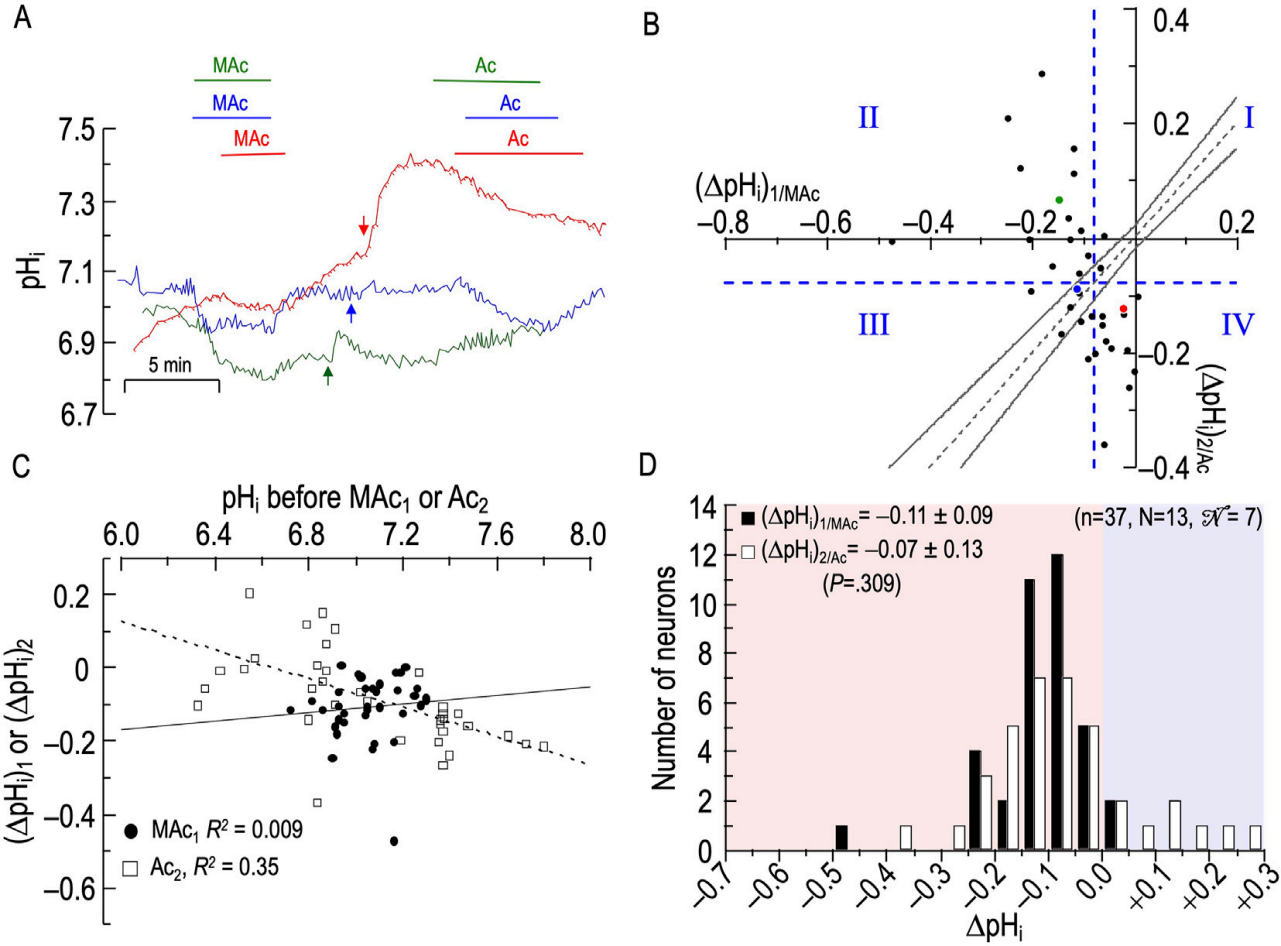
Effect of metabolic acidosis (MAc) followed by extracellular acidosis (Ac) on the pH_i_ of rat hippocampal neurons **(A)**, examples of the pH_i_ responses in three hippocampal neurons to an exposure to MAc (solution 3, Table 1) and then to Ac (solution 4, Table 1). Before the arrows, unless otherwise indicated, the bath solution was standard CO_2_/HCO_3_^−^ (solution 2, Table 1 After the arrows, unless otherwise indicated, the bath solution was our standard nominally CO_2_/HCO_3_^−^-free solution (solution 1; Table 1). **(B)**, relationship between the ΔpH_i_ during exposure to MAc—that is, (ΔpH_i_)_1/MAc_—and ΔpH_i_ during exposure to Ac—that is, (ΔpH_i_)_2/Ac_. The horizontal and vertical dashed blue lines are the resistant-sensitive demarcations that define quadrants I–IV and “pH_i_ states” (see Figure 3). The blue dot that represents the blue neuron in panel A is in Q_III_ (i.e., neuron is both MAc_1_ sensitive and Ac_2_ sensitive). The green dot is in Q_II_ (i.e., green neuron in panel **(A)** is MAc_1_ sensitive but Ac_2_ resistant). The red dot is in Q_IV_ (i.e., red neuron in panel **(A)** is MAc_1_ resistant but Ac_2_ sensitive). Note that, for the green neuron, (ΔpH_i_)_2/Ac_ is paradoxically positive. The dashed gray line is the line of identity; the gray hourglass represents the confidence interval and defines “pH_i_ behavior” (see Figure 3). Because the blue point lies within the hourglass, the behavior of the blue neuron is consistency. The green point lies above the upper asymptote of the hourglass; the behavior is adaptation. The red neuron lies below the lower asymptote of the hourglass; the behavior is decompensation. **(C)**, dependence of (ΔpH_i_)_1_ (black circles) on the initial pH_i_ before MAc_1_, which we refer to as (ΔpH_i_)_1/MAc_, or dependence of (ΔpH_i_)_2_ (white squares) on the initial pH_i_ before and Ac_2_, which we refer to as (ΔpH_i_)_2/Ac_. The solid and dashed lines represent the linear regressions for MAc_1_ and Ac_2_, respectively. **(D)**, frequency distribution of (ΔpH_i_)_1/MAc_ (black bars) and (ΔpH_i_)_2/Ac_ (white bars) with a pH_i_ bin width of 0.05 pH units. In the upper left, we report means ± standard deviation as well as the *p*-value (paired t-test, two-tails). n, number of neurons; N, number of cover slips; N , number of animals/cultures.

Figure 5A shows three examples of pH_i_ responses of HC neurons during an exposure to MAc and then to Ac. Focusing first on the blue record, we see that the neuron responds to MAc_1_ with a modest acidification. Unexpectedly, the removal of CO_2_/HCO_3_^−^ (blue arrow in Figure 5A; solution 2 → solution 1; Table 1) does not elicit the usual abrupt pH_i_ increase due to CO_2_ efflux. It is possible that strong AE3 activity (Salameh et al., 2017) produced a HCO_3_^−^ efflux that nullified the pH_i_ effects of CO_2_ efflux. In the continued absence of CO_2_/HCO_3_^−^, imposing Ac_2_ produces a slow but sustained pH_i_ decrease. It is not clear whether this pH_i_ decrease would have subsided, had we extended the Ac exposure.^8^ Restoring pH_o_ 7.40 produces the expected pH_i_ recovery.

The neuron represented by the green record has a larger response to MAc_1_, a small pH_i_ recovery after removal of MAc, and a modest pH_i_ increase due to CO_2_ efflux upon removal of CO_2_/HCO_3_^−^. Ac_2_ paradoxically causes a frank alkalinization. The red record slowly approaches a stable pH_i_ in CO_2_/HCO_3_^−^, and MAc_1_ elicits only a small pH_i_ decrease, the magnitude of which we may have slightly underestimated because pH_i_ may not have been entirely stable before the MAc_1_. Nevertheless, the return to pH_o_ 7.40 in CO_2_/HCO_3_^−^ elicits a robust pH_i_ increase. The subsequent removal of CO_2_/HCO_3_^−^ produces a very large pH_i_ increase (perhaps reflecting a low AE3 activity), and the Ac_2_ challenge leads to a slow, seemingly interminable pH_i_ decrease. Removing Ac_2_ does not elicit a pH_i_ recovery, at least in the brief time allotted. We present these Ac_2_ data so that the reader may be aware that pH_o_ changes under non-physiological conditions often lead to unexpected consequences.

#### Analyses #1–3

Figure 5B and Table 5 summarize state and behavior for the full MAc-Ac dataset (n = 37, N = 13, N = 7). Figure 5B reveals a very different pattern from Figure 4B, with many neurons deviating markedly from the hourglass. Most neurons lie in band that runs from the upper-left (Q_II_) to the lower-right (Q_IV_), and thus range in behavior from strong adaptation to strong decompensation.

**TABLE 5:** State and behavior of 37 hippocampal neurons during exposure to metabolic acidosis (MAc) and extracellular acidosis (Ac).

A, state:* MAc_1_	16 neurons (43%) = resistant		21 neurons (57%) = sensitive		
B, state transition:† MAc_1_→ Ac_2_	3 (8%) remain resistant [Q_I_]	13 (35%) become sensitive [Q_IV_]	9 (24%) become resistant [Q_II_]	12 (33%) remain sensitive [Q_III_]	
C, Behavior:‡ MAc_1_→ Ac_2_*					Total
Adaptation	1 (3%)	0	9 (24%)	6 (16%)	16 (43%)
Consistency	2 (5%)	0	0	4 (11%)	6 (16%)
Decompensation	0	13 (35%)	0	2 (6%)	15 (41%)
D, State:† Ac_2_ Resistant	12 (32%)		Ac_2_ Sensitive		25 (66%)

^a^ State for pulse 1: resistant neurons are to the right of the vertical dashed blue line; sensitive neurons are to the left.

^b^ State for pulse 2, resistant neurons are above the horizontal dashed blue line; sensitive neurons are below.

^c^ Behaviors for pulse 1→2 transition: Adaptation (i.e., point is above the hourglass); Consistency (i.e., point is within the hourglass); Decompensation (i.e., point is below the hourglass).

Q, quadrant; Q_I_, quadrant I (neurons that are resistant during MAc_1_, and remain “resistant” during Ac_2_); Q_II_, quadrant II (MAc_1_ sensitive → Ac_2_ resistant); Q_III_, quadrant III (MAc_1_ sensitive → Ac_2_ sensitive); Q_IV_, quadrant IV (MAc_1_ resistant → Ac_2_ sensitive).

**Key points:** (1) Of the 16 MAc_1_-resistant neurons (row A), 13 fulfill the criteria for decompensation in the transition to Ac_2_. (2) Of the 16 neurons with an adaptation behavior in the transition to Ac_2_ (row C), 15—including all eight neurons with a paradoxical alkalinizing response in Ac_2_—were MAc_1_ sensitive.

The blue point lies in Q_III_ (i.e., states are MAc_1_ and Ac_2_ sensitive), and within the hourglass (i.e., behavior is consistency).

The green point lies in Q_II_ (i.e., states are MAc_1_ sensitive but Ac_2_ resistant), and well above the hourglass (i.e., behavior is adaptation). In fact, this is one of eight neurons that fall at least 0.02 above the *x*-axis. This frequency of frank alkalinization during Ac_2_ (8 of 37 neurons = 19%) is far higher than for Ac_1_ in Figure 4B (2 of 39 = 5% are to the right of the *y*-axis). Thus, the MAc_1_ pretreatment promotes paradoxical alkalinization during Ac_2_—a theme that repeats itself below when MAc_1_ precedes pAc_2_ or pMet↓_2_. Because the extracellular solution is nominally free of CO_2_/HCO_3_^−^, Na-H exchange most likely mediates this paradoxical alkalization, to the extent that it is opposed by background acid loading (Bevensee and Boron, 2013).

The red point in Figure 5B lies in Q_IV_ (i.e., states are MAc_1_ resistant but Ac_2_ sensitive), and below the hourglass (i.e., behavior is decompensation).

The *d*_±_ of +0.023 (Table 2, row 3) reveals a trend toward adaptation. However, this is not significantly different from zero because the SD of 0.138 is so large, consistent with our subjective impression of a broad dispersion of neurons from Q_II_ to Q_IV_.

#### ΔpH_i_ vs initial pH_i_

Figure 5C reveals a correlation strength, taking all 52 points together, of “absent” between (ΔpH_i_)_1/MAc_ and (pH_i_)_1_. The weak correlation strength between (ΔpH_i_)_2/Ac_ and (pH_i_)_2_ is consistent with a greater acidification for neurons with a higher pre-Ac_2_ pH_i_.

#### Frequency distributions

Figure 5D shows the frequency distributions of ΔpH_i_ for both MAc_1_ and Ac_2_. Although the Ac_2_ distribution tends to shift to the right, the difference is not statistically significant.

#### Summary of MAc-Ac

With some neurons, Ac_2_ causes seemingly continuous pH_i_ decreases and strong decompensating behavior. In others, perhaps primed by the preceding MAc_1_, Ac_2_ leads to strong adaptive behavior and often frank, paradoxical alkalinization.

### An isolated pH_o_ decrease (pAc) and then MAc

A limitation of the Ac-MAc and MAc-Ac studies is that the absence of CO_2_/HCO_3_^−^ during the fall of pH_o_ could limit both the sensor and effector arms of any cellular response. To gain further understanding on the behavior of HC neurons during extracellular acid-base challenges, in the remainder of the present paper we exploit CO_2_/HCO^−^_3_ out-of-equilibrium solutions to modify only one parameter of the Henderson-Hasselbalch equation. First, we use OOE technology to lower pH_o_ to 7.20—the same acidification that we achieve during MAc—while maintaining a stable [CO_2_]_o_ and [HCO^−^_3_]_o_. This pure acidosis protocol begins with neurons bathed in our standard CO_2_/HCO_3_^−^ solution, followed by challenges first with pAc (solution 5, Table 1) and then with MAc.

#### Sample pH_i_ records

**FIGURE 6:**
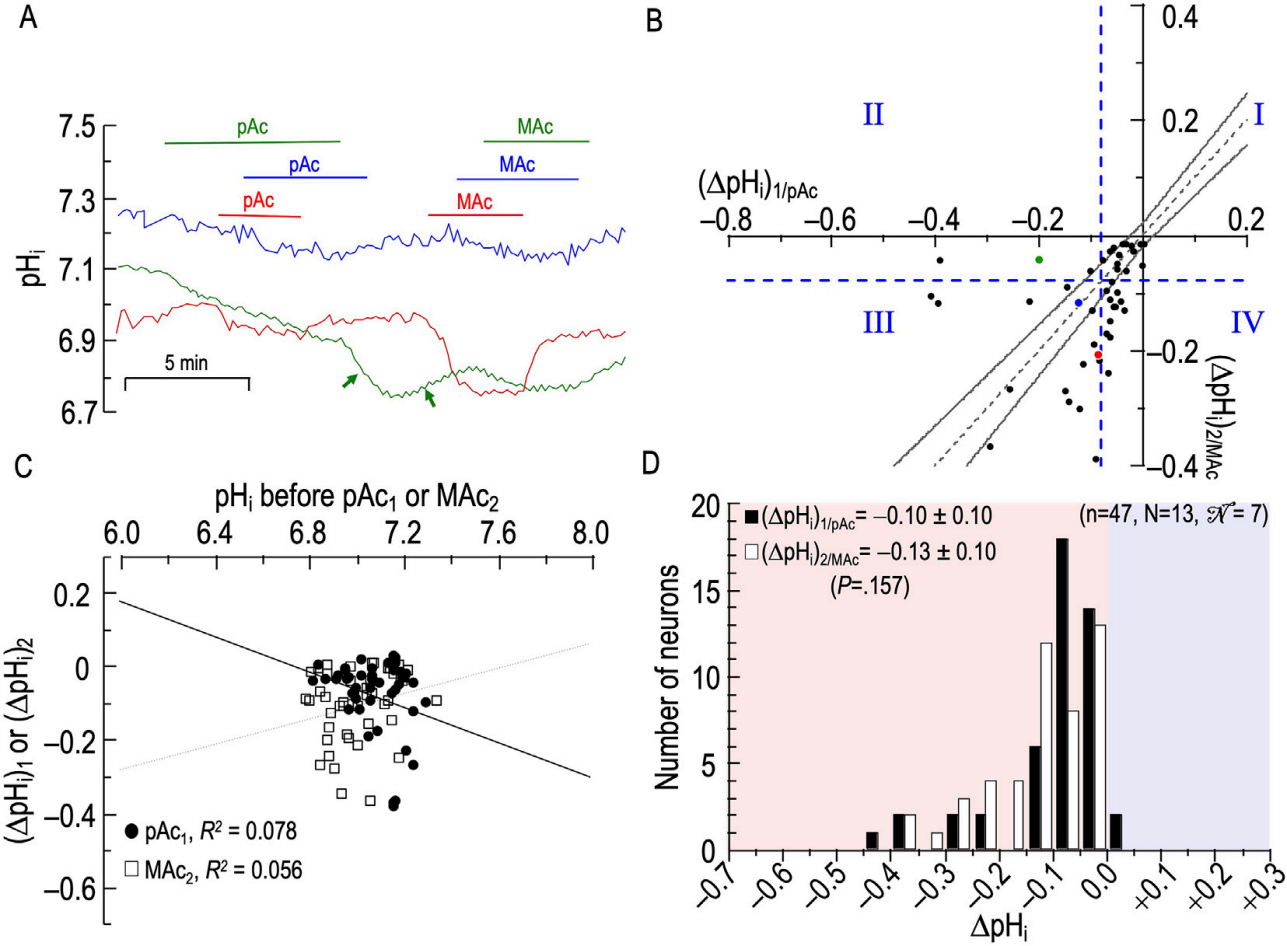
Effect of an isolated decrease of pH_o_ (pAc) followed by metabolic acidosis (MAc) on the pH_i_ of rat hippocampal neurons **(A)**, examples of the pH_i_ responses in three hippocampal neurons to an exposure to pAc (solution 5, Table 1), and then to MAc (solution 3, Table 1). Unless otherwise indicated, the bath solution was standard CO_2_/HCO_3_^−^ (solution 2, Table 1). The green arrows following pAc removal indicate an initial anomalous pH_i_ decrease (left) followed by the expected but delayed pH_i_ increase (right). **(B)**, relationship between the ΔpH_i_ during exposure to pAc—that is, (ΔpH_i_)_1/pAc_—and ΔpH_i_ during exposure to MAc—that is, (ΔpH_i_)_2/MAc_. The horizontal and vertical dashed blue lines are the resistant-sensitive demarcations that define quadrants I–IV and “pH_i_ states” (see Figure 3). The blue dot that represents the blue neuron in panel A is in Q_III_ (i.e., neuron is both pAc_1_ sensitive and MAc_2_ sensitive). The green dot is in Q_II_ (i.e., green neuron in panel A is pAc_1_ sensitive but MAc_2_ resistant). Because the red dot is slightly to the left of the vertical dashed blue line, it is in Q_IV_ (i.e., red neuron in panel A is both pAc_1_ sensitive and MAc_2_ sensitive). The dashed gray line is the line of identity; the gray hourglass represents the confidence interval and defines “pH_i_ behavior” (see Figure 3). Because the blue point lies within the hourglass, the behavior of the blue neuron is consistency. The green point lies above the upper asymptote of the hourglass; the behavior is adaptation. The red neuron lies below the lower asymptote of the hourglass; the behavior is decompensation. **(C)**, dependence of (ΔpH_i_)_1_ (black circles) on the initial pH_i_ before pAc_1_, which we refer to as (ΔpH_i_)_1/pAc_, or dependence of (ΔpH_i_)_2_ (white squares) on the initial pH_i_ before and MAc_2_, which we refer to as (ΔpH_i_)_2/MAc_. The solid and dashed lines represent the linear regressions for pAc_1_ and MAc_2_, respectively. **(D)**, frequency distribution of (ΔpH_i_)_1/pAc_ (black bars) and (ΔpH_i_)_2/MAc_ (white bars) with a pH_i_ bin width of 0.05 pH units. In the upper left, we report means ± standard deviation as well as the *p*-value (paired t-test, two-tails). N, number of neurons; N, number of cover slips; N , number of animals/cultures.

Figure 6A shows three pH_i_ responses of HC neurons. The blue trace represents a neuron with modest acidification responses to pAc and MAc.

The green trace represents a neuron in which pAc_1_ elicits a modestly rapid pH_i_ decline that shows no sign of abating. The removal of the pAc challenge, rather than heralding a pH_i_ recovery, initially produces an even more rapid acidification (first green arrow); we observed this pattern, though less dramatically, in four other neurons. Eventually, pH_i_ spontaneously reverses direction and rises fairly rapidly (second green arrow); we observed this pattern only in this neuron. Finally, the subsequent MAc_2_ challenge in the green neuron now elicits only a small pH_i_ decrease. It is possible that the spontaneous reversal of the pH_i_ decline (green arrow) is adaption in the making.

Finally, the red trace represents a neuron that displays a small acidification during pAc_1_ but acidifies markedly during the subsequent MAc_2_.

#### Analyses #1–3

Figure 6B and Table 6 summarize state and behavior for the full pAc-MAc dataset (n = 47, N = 13, N = 7). Two characteristics of Figure 6B are noteworthy. First, most of the points lie to the right of the vertical dashed blue line. That is, during pAc_1_, most neurons fulfill the MAc_1_ criterion for a resistant state. Second, most points lie barely above, within, or below the hourglass. That is, their behavior tends to be consistency or decompensation. Very few neurons (e.g., the green point) display adaptation behavior, and none lie above the *x*-axis.

**TABLE 6:** State and behavior of 47 hippocampal neurons during exposure to isolated decrease of pH_o_ (pAc) and metabolic acidosis (MAc).

A, state:* pAc_1_	27 neurons (57%) = resistant		20 neurons (43%) = sensitive		
B, state transition:† pAc_1_→ MAc_2_	15 (32%) remain resistant [Q_I_]	12 (25%) become sensitive [Q_IV_]	3 (7%) become resistant [Q_II_]	17 (36%) remain sensitive [Q_III_]	
C, Behavior:‡ pAc_1_→ MAc_2_*					Total
Adaptation	2 (4%)	0	3 (7%)	4 (9%)	9 (19%)
Consistency	10 (21%)	1 (2%)	0	2 (4%)	13 (28%)
Decompensation	3 (7%)	11 (23%)	0	11 (23%)	25 (53%)
D, State:† MAc_2_ Resistant	18 (38%)		MAc_2_ Sensitive	29 (62%)	

^a^ State for pulse 1: resistant neurons are to the right of the vertical dashed blue line; sensitive neurons are to the left.

^b^ State for pulse 2, resistant neurons are above the horizontal dashed blue line; sensitive neurons are below.

^c^ Behaviors for pulse 1→2 transition: Adaptation (i.e., point is above the hourglass); Consistency (i.e., point is within the hourglass); Decompensation (i.e., point is below the hourglass).

Q, quadrant; Q_I_, quadrant I (neurons that are “resistant” during pAc_1_, and remain resistant during MAc_2_); Q_II_, quadrant II (pAc_1_ sensitive → MAc_2_ resistant); Q_III_, quadrant III (pAc_1_ sensitive → pAc_2_ sensitive); Q_IV_, quadrant IV (pAc_1_ resistant → MAc_2_ sensitive).

**Key points:** The general trend is toward MAc_2_ a sensitivity: Of 27 neurons with a resistant state during pAc_1_ (row A), nearly half adopt a sensitive (12; Q_IV_) state during MAc_2_ (row A). Of 20 neurons with a sensitive state during pAc_1_, the vast majority (17; Q_III_) adopt a sensitive state during MAc_2_.

The blue point lies in Q_III_ (i.e., states are pAc_1_ and MAc_2_ sensitive), and nearly on the gray dashed line within the hourglass (i.e., behavior is consistency).

The green point lies in Q_II_ (i.e., states are pAc_1_ sensitive but MAc_2_ resistant), and well above the hourglass (i.e., behavior is adaptation).

The red point lies in Q_III_, just to the left of the vertical dashed blue line (i.e., states are barely pAc_1_ sensitive but solidly MAc_2_ sensitive), and below the hourglass (i.e., behavior is decompensation).

Although the mean *d*_±_ of −0.015 (Table 2, row 4) trends toward decompensation, this value is not significantly different from zero.

#### **Δ**pH_i_ vs initial pH_i_

Figure 6C reveals correlation strengths, taking all 47 points together, of “absent” both between (ΔpH_i_)_1/pAc_ and (pH_i_)_1_, and between (ΔpH_i_)_2/MAc_ and (pH_i_)_2_.

#### Frequency distributions

Figure 6D summarizes the distribution of ΔpH_i_ during the two conditions, and shows that, although we see a tendency for a leftward shift during the second challenge, (ΔpH_i_)_2/MAc_ is not significantly different from(ΔpH_i_)_1/pAc_.

#### Summary of pAc-MAc

pAc_1_ generally produces a small acidification (resistant “state”), followed during MAc_2_ by a trend toward decompensation.

### MAc and then pAc

In this experimental series, we invert the sequence of exposure from the previous protocol, challenging neurons first with MAc then with pAc.

### Sample pH_i_ records

**FIGURE 7:**
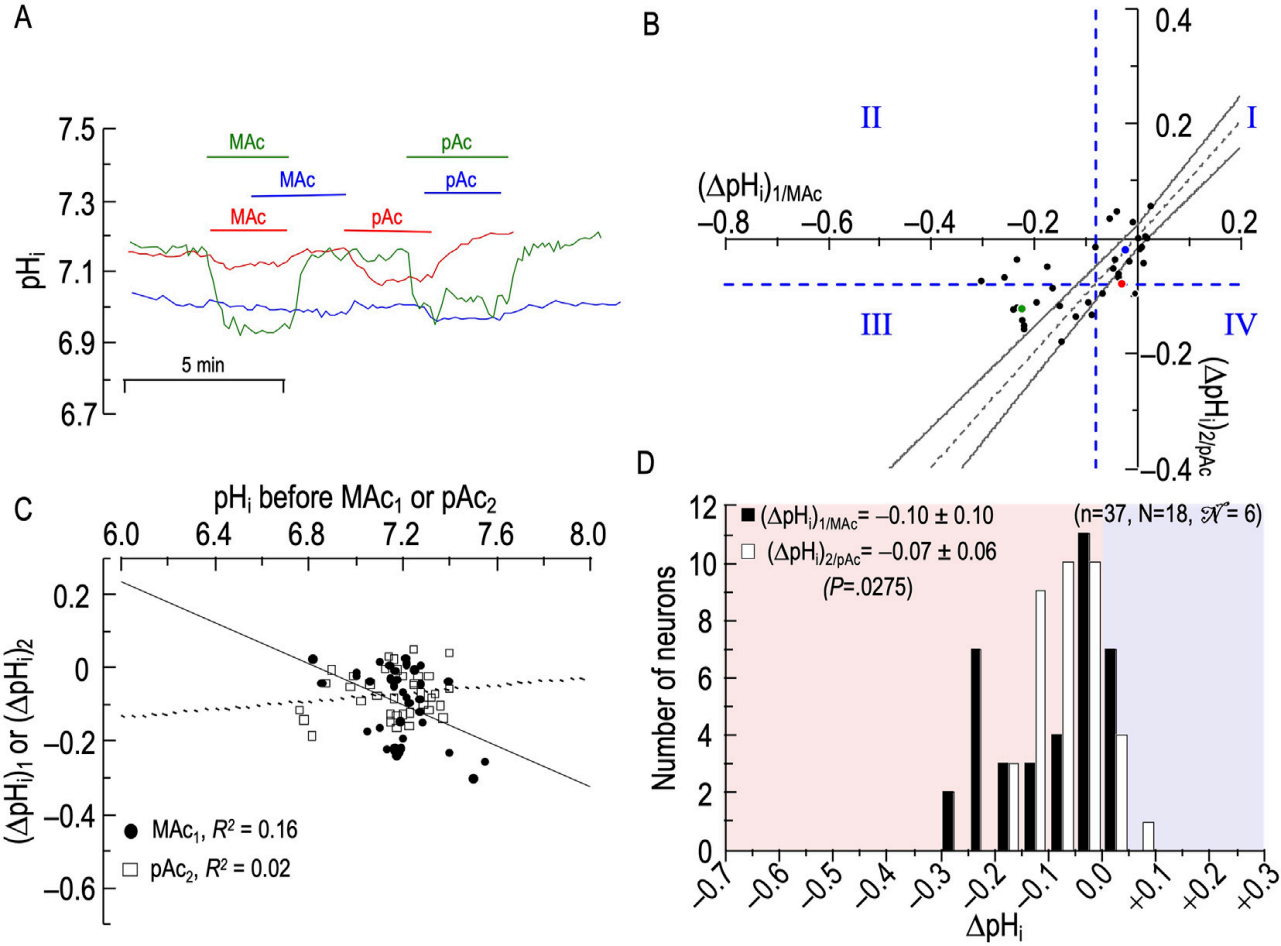
Effect of metabolic acidosis (MAc) followed by pure acidosis (pAc) on the pH_i_ of rat hippocampal neurons **(A)**, examples of the pH_i_ responses in three hippocampal neurons to an exposure to MAc (solution 3, Table 1), and then to pAc (solution 5, Table 1). Unless otherwise indicated, the bath solution was standard CO_2_/HCO_3_^−^ (solution 2, Table 1). **(B)**, relationship between the ΔpH_i_ during exposure to MAc—that is, (ΔpH_i_)_1/MAc_—and ΔpH_i_ during exposure to pAc—that is, (ΔpH_i_)_2/pAc_. The horizontal and vertical dashed blue lines are the resistant-sensitive demarcations that define quadrants I–IV and “pH_i_ states” (see Figure 3). The blue dot that represents the blue neuron in panel A is in Q_I_ (i.e., neuron is both MAc_1_ and pAc_2_ resistant). The green dot is in Q_III_ (i.e., green neuron in panel A is both MAc_1_ and pAc_2_ sensitive). Because the red dot is on the horizontal dashed blue line, it is in Q_I_ (i.e., red neuron in panel A is both MAc_1_ and pAc_2_ resistant). The dashed gray line is the line of identity; the gray hourglass represents the confidence interval and defines “pH_i_ behavior” (see Figure 3). Because the blue point lies within the hourglass, the behavior of the blue neuron is consistency. The green point lies above the upper asymptote of the hourglass; the behavior is adaptation. The red neuron lies below the lower asymptote of the hourglass; the behavior is decompensation. **(C)**, dependence of (ΔpH_i_)_1_ (black circles) on the initial pH_i_ before MAc_1_, which we refer to as (ΔpH_i_)_1/MAc_, or dependence of (ΔpH_i_)_2_ (white squares) on the initial pH_i_ before and pAc_2_, which we refer to as (ΔpH_i_)_2/pAc_. The solid and dashed lines represent the linear regressions for MAc_1_ and pAc_2_, respectively. **(D)**, frequency distribution of (ΔpH_i_)_1/MAc_ (black bars) and (ΔpH_i_)_2/pAc_ (white bars) with a pH_i_ bin width of 0.05 pH units. In the upper left, we report means ± standard deviation as well as the *p*-value (paired t-test, two-tails). n, number of neurons; N, number of cover slips; N , number of animals/cultures.

Figure 7A shows three pH_i_ responses of HC neurons. The blue pH_i_ trace represents a neuron that exhibits almost no change in pH_i_ during MAc_1_ exposure, and acidifies only slightly during pAc_2_. The green trace reflects large acidifications during both MAc_1_ and, to a lesser extent, pAc_2_. Finally, the red pH_i_ trace reports only a small acidification during MAc_1_ but a much larger one during pAc_2_.

#### Analyses #1–3

Figure 7B and Table 7 summarize state and behavior for the full MAc-pAc dataset (n = 37, N = 18, N = 6). In contrast to pAc-MAc, for which Figure 6B gives the subjective impression of many neurons lying below the hourglass, MAc-pAc gives a different impression, with most points line in or near the hourglass ∼40% of neurons lying above the hourglass (i.e., adaptation “behavior”).

**TABLE 7:** State and behavior of 37 hippocampal neurons behavior during metabolic acidosis (MAc) and to an isolated decrease of pH_o_ (pAc) exposure.

A, state:* MAc_1_	20 neurons (54%) = resistant		17 neurons (46%) = sensitive		
B, state transition:† MAc_1_→ pAc_2_	18 (49%) remain resistant [Q_I_]	2 (5%) become sensitive [Q_IV_]	4 (11%) become resistant [Q_II_]	13 (35%) remain sensitive [Q_III_]	
C, Behavior:‡ MAc_1_→ pAc_2_*					Total
Adaptation	4 (11%)	0	4 (11%)	8 (22%)	16 (43%)
Consistency	5 (14%)	0	0	3 (8%)	8 (22%)
Decompensation	9 (24%)	2 (5%)	0	2 (5%)	13 (35%)
D, State:† pAc_2_ Resistant	22 (60%)		pAc_2_ Sensitive	15 (40%)	

^a^ State for pulse 1: resistant neurons are to the right of the vertical dashed blue line; sensitive neurons are to the left.

^b^ State for pulse 2, resistant neurons are above the horizontal dashed blue line; sensitive neurons are below.

^c^ Behaviors for pulse 1→2 transition: Adaptation (i.e., point is above the hourglass); Consistency (i.e., point is within the hourglass); Decompensation (i.e., point is below the hourglass).

Q, quadrant; Q_I_, quadrant I (neurons that are resistant during MAc_1_, and remain “resistant” during MAc_2_); Q_II_, quadrant II (MAc_1_ sensitive → pAc_2_ resistant); Q_III_, quadrant III (MAc_1_ sensitive → pAc_2_ sensitive); Q_IV_, quadrant IV (MAc_1_ resistant → pAc_2_ sensitive).

**Key points:** The neurons tend to maintain their “state” between MAc_1_ (row A) and pAc_2_ (row B). Thus, 18 of 20 neurons that were MAc_1_ resistant also fulfill the MAc, criteria for resistance during pAc_2_ (Q_I_), and 13 of 17 neurons that were MAc_1_ sensitive also fulfill the MAc, criteria for sensitivity in pAc_2_ (Q_III_). We suggest that this MAc_1_–pAc_2_ comparison is valid because our global (ΔpH_i_)_1/MAc_, of −0.11 in Figure 1 is very similar to the (ΔpH_i_)_1/pAc_ of −0.10 in the pAc-MAc, protocol (Figure 6D). Although nearly 85% of the neurons lie in Q_I_, or Q_III_, only a small fraction (22%) of MAc-pAc neurons fulfill the MAc-MAc, criteria for consistency.

The blue point lies in Q_I_ (i.e., states are both MAc_1_ and pAc_2_ resistant), and virtually on the LOI within the hourglass (i.e., behavior is consistency).

The green point lies in Q_III_ (i.e., states are both MAc_1_ and pAc_2_ sensitive), and above the hourglass (i.e., behavior is adaptation).

The red point lies on the Q_I_/Q_IV_ boundary (i.e., states are MAc_1_ resistant and barely pAc_2_ resistant), and just below the hourglass (i.e., behavior is decompensation).

Consistent with our subjective impression of a predominance of adaptation (i.e., a plurality of neurons lying above the hourglass), the mean *d*_±_ is +0.020 (Table 2, row 5), a value significantly different from zero (*p* = .0275).

#### **Δ**pH_i_ vs initial pH_i_

Figure 7C shows correlation strengths, taking all 37 points together, of “absent”, both for (ΔpH_i_)_1_ vs (pH_i_)_1_, and for (ΔpH_i_)_2_ vs (pH_i_)_1_.

#### Frequency distributions

Figure 7D shows that the mean (ΔpH_i_)_2/pAc_ is less than the mean (ΔpH_i_)_1/MAc_, and that the difference is statistically significant.

#### Summary of MAc-pAc

Following MAc_1_, pAc_2_ tends to produce relatively small acidifications.

### An isolated decrease in [HCO_3_^−^]_o_ (pMet↓) and then MAc

In the two previous experimental series, we explored the effects of an isolated decrease of pH_o_—one component of MAc—on neuronal pH_i_. In this and the next section, we investigate the effects of the other component of MAc, an isolated decrease of [HCO_3_^−^]_o_. Our approach is to use an OOE CO_2_/HCO^−^_3_ solution to keep [CO_2_]_o_ and pH_o_ constant as we lower [HCO_3_^−^]_o_ to the same extent as we would in a MAc solution. This pure metabolic/decreasing [HCO_3_^−^]_o_ (pMet↓) protocol begins with neurons bathed in our standard CO_2_/HCO_3_^−^ solution, followed by two challenges, pMet↓ (solution 6, Table 1) and then MAc.

#### Sample pH_i_ records

**FIGURE 8:**
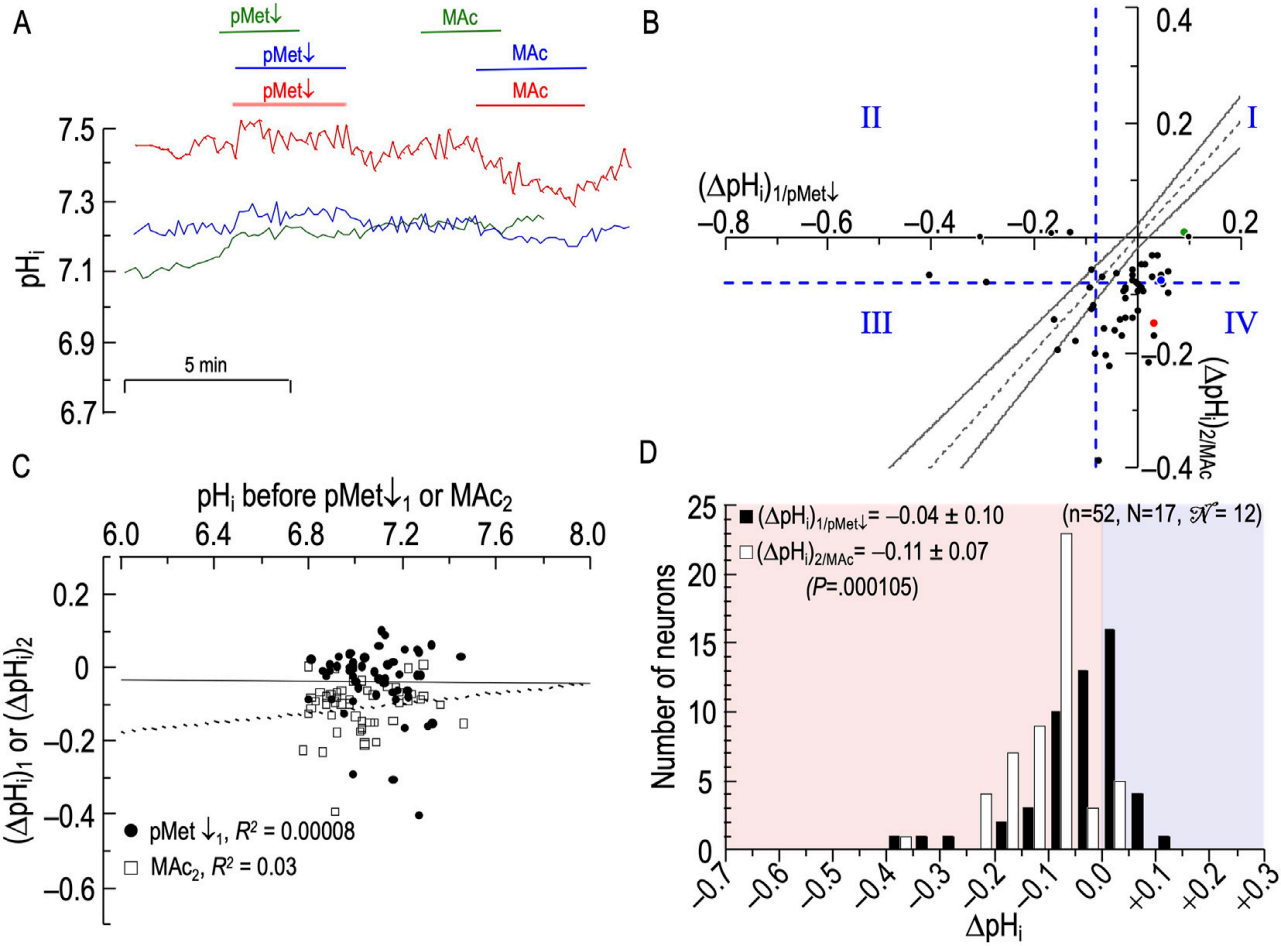
Effect of an isolated decrease of [HCO_3_^−^]_o_ (pMet↓) followed by metabolic acidosis (MAc) on the pH_i_ of rat hippocampal neurons **(A)**, examples of the pH_i_ responses in three hippocampal neurons to an exposure to pMet↓ (solution 6, Table 1), and then to MAc (solution 3, Table 1). Unless otherwise indicated, the bath solution was standard CO_2_/HCO_3_^−^ (solution 2, Table 1). **(B)**, relationship between the ΔpH_i_ during exposure to pMet↓—that is, (ΔpH_i_)_1/pMet↓_—and ΔpH_i_ during exposure to MAc—that is, (ΔpH_i_)_2/MAc_. The horizontal and vertical dashed blue lines are the resistant-sensitive demarcations that define quadrants I–IV and “pH_i_ states” (see Figure 3). The blue dot that represents the blue neuron in panel **(A)** is in Q_I_ (i.e., neuron is both pMet↓ and MAc_2_ resistant). The green dot also is in Q_I_ (i.e., green neuron in panel **(A)** is both pMet↓ and MAc_2_ resistant). The red dot is in Q_IV_ (i.e., red neuron in panel **(A)** is pMet↓_1_ resistant but MAc_2_ sensitive). Note that, for all three neurons, (ΔpH_i_)_1/pMet↓_ is paradoxically positive. Additionally, for the green neuron, (ΔpH_i_)_2/MAc_ is positive. The dashed gray line is the line of identity; the gray hourglass represents the confidence interval and defines “pH_i_ behavior” (see Figure 3). Because the blue, green, and red points all lie below the lower asymptote of the hourglass, the behavior of the three corresponding neurons is decompensation. **(C)**, dependence of (ΔpH_i_)_1_ (black circles) on the initial pH_i_ before pMet↓, which we refer to as (ΔpH_i_)_1/pMet↓_, or dependence of (ΔpH_i_)_2_ (white squares) on the initial pH_i_ before and MAc_2_, which we refer to as (ΔpH_i_)_2/MAc_. The solid and dashed lines represent the linear regressions for pMet↓_1_ and MAc_2_, respectively. **(D)**, frequency distribution of (ΔpH_i_)_1/pMet↓_ (black bars) and (ΔpH_i_)_2/MAc_ (white bars) with a pH_i_ bin width of 0.05 pH units. In the upper left, we report means ± standard deviation as well as the *p*-value (paired t-test, two-tails). n, number of neurons; N, number of cover slips; N , number of animals/cultures.

Figure 8A shows three responses of HC neurons. The blue trace represents a neuron with a small paradoxical alkalinization in response to pMet↓_1_—paradoxical because an isolated decrease in [HCO_3_^−^]_o_ elicits a rise in pH_i_ and thus (because [CO_2_]_i_ ≅ [CO_2_]_o_) a rise in [HCO_3_^−^]_i_—and a small acidification in response to MAc_2_. The green trace also reports a paradoxical alkalinization during pMet↓_1_, followed by almost no change during MAc_2_. Finally, the red trace again reveals a paradoxical alkalinization during pMet↓_1_, but then a marked acidification during MAc_2_.

#### Analyses #1–3

Figure 8B and Table 8 summarize the state and behavior for the full pMet↓-MAc dataset (n = 52; N = 17, N = 12). Because pH_o_ remains constant during pMet↓_1_, the ratio ΔpH_i_/ΔpH_o_ = ±∞ (or undefined in the case of 0/0) for each neuron. Although it is impossible to position the vertical dashed blue line at ΔpH_i_ = 40% × pH_o_, we elect to position it at (ΔpH_i_)_1/pMet↓_ = 0.08, the value for the parent disturbance, (ΔpH_i_)_1/MAc_.

**TABLE 8:** State and behavior of 52 hippocampal neurons behavior during an isolated decrease of [HCO^−^_3_]_o_ (pMet↓) and MAc exposure.

A, state:* pMet↓_1_	40 neurons (77%) = resistant		12 neurons (23%) = sensitive		
B, state transition:† pMet↓_1_ → MAc_2_	16 (31%) remain resistant [Q_I_]	24 (46%) become sensitive [Q_IV_]	6 (11.5%) become resistant [Q_II_]	6 (11.5%) remain sensitive [Q_III_]	
C, Behavior:‡ pMet↓_1_ → MAc_2_*					Total
Adaptation	1 (2%)	0	5 (9%)	0	6 (11%)
Consistency	1 (2%)	0	1 (2%)	2 (4%)	4 (8%)
Decompensation	14 (27%)	24 (46%)	0	4 (8%)	42 (81%)
D, State:† MAc_2_ Resistant	22 (42%)		MAc_2_ Sensitive	30 (58%)	

*State for pulse 1: resistant neurons are to the right of the vertical dashed blue line; sensitive neurons are to the left.

†State for pulse 2, resistant neurons are above the horizontal dashed blue line; sensitive neurons are below.

‡Behaviors for pulse 1→2 transition: Adaptation (i.e., point is above the hourglass); Consistency (i.e., point is within the hourglass); Decompensation (i.e., point is below the hourglass).

Q, quadrant; Q_I_, quadrant I (neurons that are “resistant” during pMet↓_1_, and remain resistant during MAc_2_); Q_II_, quadrant II (pMet↓_1_ sensitive → MAc_2_ resistant); Q_III_, quadrant III (pMet↓_1_ sensitive → MAc_2_ sensitive); Q_IV_, quadrant IV (pMet↓_1_ resistant → MAc_2_ sensitive).

**Key points:** Most neurons (40 of 52) are resistant during pMet↓_1_ by MAc, criteria (row A). Of these, most (24 of 40) are sensitive during MAc_2_ (row B). Of the few neurons (12 of 52) that are pMet↓_1_ sensitive, equal numbers are resistant vs sensitive during MAc_2_. The rightmost column of the table reveals that >80% of the neurons fulfill the criteria for decompensation.

The most striking characteristic of Figure 8B is that the vast majority of points fall to the right of the vertical dashed blue line, and all but five of 52 neurons lie in or below the hourglass. In other words, in a naïve neuron, pMet↓ produces only small pH_i_ decreases or even paradoxical pH_i_ increases, and the subsequent MAc_2_ nearly always produces larger pH_i_ decreases that fulfill the criteria for decompensation behavior.

The blue point lies in Q_I_ (i.e., states are both pMet↓_1_ and MAc_2_ resistant), well below the hourglass (i.e., behavior is decompensation), and lies to the right of the *y*-axis (i.e., pMet↓_1_ elicits a paradoxical alkalinization).

The green point, like the blue point, lies in Q_I_ (i.e., states are both pMet↓_1_ and MAc_2_ resistant) and is below the hourglass (i.e., behavior is decompensation). It is not only to the right of the *y*-axis, it is just above the *x*-axis (i.e., pMet↓_1_ elicits a strong paradoxical alkalinization).

The red point lies in Q_IV_ (i.e., states are pMet↓_1_ resistant but MAc_2_ sensitive), and well below the hourglass (i.e., behavior is decompensation).

The mean *d*_±_ is −0.047 (Table 2, row 6), the most strongly negative value (consistent with decompensation) in the present study; this value is significantly different from zero (*P* ≅ 0.0001).

#### **Δ**pH_i_ vs initial pH_i_

Figure 8C reveals correlation strengths, taking all 52 points together, of “absent” for both (ΔpH_i_)_1/pMet↓_ vs (pH_i_)_1_, and (ΔpH_i_)_2/MAc_ vs (pH_i_)_2_.

#### Frequency distributions

Figure 8D shows that the mean (ΔpH_i_)_1/pMet↓_ is substantially less than the mean for (ΔpH_i_)_2/MAc_, and that the difference is highly significant.

#### Summary of pMet**↓**-MAc

pMet↓_1_ produces the smallest pH_i_ decrease of any first challenge in the present study, and often produces paradoxical pH_i_ increases.

### MAc and then pMet↓

In this final experimental series, we reverse the order of pMet↓ and MAc from Figure 8, challenging the neurons first with MAc, and then with pMet↓.

#### Sample pH_i_ records

**FIGURE 9:**
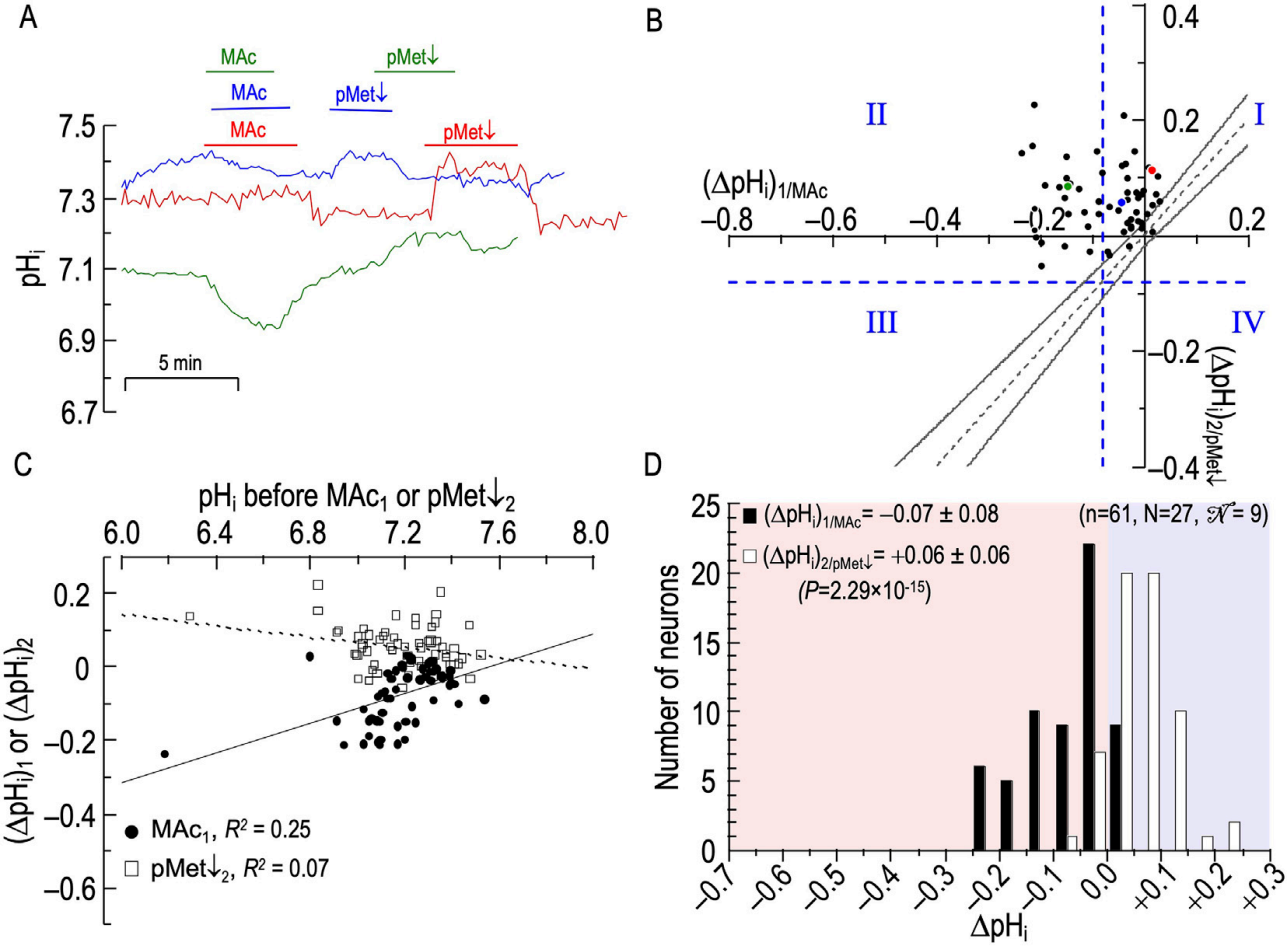
Effect of metabolic acidosis (MAc) followed by pure metabolic/down (pMet↓) on the pH_i_ of rat hippocampal neurons **(A)**, examples of the pH_i_ responses in three hippocampal neurons to an exposure to MAc (solution 3, Table 1), and then to pMet↓ (solution 6, Table 1). Unless otherwise indicated, the bath solution was standard CO_2_/HCO_3_^−^ (solution 2, Table 1). **(B)**, relationship between the ΔpH_i_ during exposure to MAc—that is, (ΔpH_i_)_1/MAc_—and ΔpH_i_ during exposure to pMet↓—that is, (ΔpH_i_)_2/pMet↓_. The horizontal and vertical dashed blue lines are the resistant-sensitive demarcations that define quadrants I–IV and “pH_i_ states” (see Figure 3). The blue dot that represents the blue neuron in panel **(A)** is in Q_I_ (i.e., neuron is both pMet↓ and MAc_2_ resistant). The green dot is in Q_II_ (i.e., green neuron in panel **(A)** is MAc_1_ sensitive but pMet↓_2_ resistant). The red dot is in Q_I_ (i.e., red neuron in panel **(A)** is both MAc_1_ pMet↓_2_ resistant). Note that, for all three neurons, (ΔpH_i_)_2/pMet↓_ is paradoxically positive. Additionally, for the red neuron, (ΔpH_i_)_1/MAc_ is positive. The dashed gray line is the line of identity; the gray hourglass represents the confidence interval and defines “pH_i_ behavior” (see Figure 3). Because the blue, green, and red points are all above the upper asymptote of the hourglass, the behavior is adaptation. **(C)**, dependence of (ΔpH_i_)_1_ (black circles) on the initial pH_i_ before MAc_1_, which we refer to as (ΔpH_i_)_1/MAc_, or dependence of (ΔpH_i_)_2_ (white squares) on the initial pH_i_ before and pMet↓_2_, which we refer to as (ΔpH_i_)_2/pMet↓_. The solid and dashed lines represent the linear regressions for Ac_1_ and MAc_2_, respectively. **(D)**, frequency distribution of (ΔpH_i_)_1/MAc_ (black bars) and (ΔpH_i_)_2/pMet↓_ (white bars) with a pH_i_ bin width of 0.05 pH units. In the upper left, we report means ± standard deviation as well as the *p*-value (paired t-test, two-tails). n, number of neurons; N, number of cover slips; N , number of animals/cultures.

Figure 9A shows the remarkable pH_i_ responses of three HC neurons. The blue record is that of a neuron that acidifies slightly in response to MAc_1_, hardly recovers upon withdrawal of the MAc_1_ challenge, and then paradoxically alkalinizes during pMet↓_2_. The green trace shows a large acidification during MAc_1_ but a modest, paradoxical alkalinization with pMet↓_2_. Finally, the red trace reports a small, paradoxical alkalinization in response to MAc_1_, a paradoxical acidification upon removal of the MAc_1_ challenge, and a large, paradoxical alkalinization during pMet↓_2_.

#### Analyses #1–3

Figure 9B and Table 9 summarize state and behavior for the full MAc-pMet↓ dataset (n = 61; N = 24, N = 9). As noted for pMet↓_1_ in Figure 8B, during pMet↓_2_ here in Figure 9B, ΔpH_i_/ΔpH_o_ = ±∞ (or 0/0). Therefore, we elect to position the horizontal dashed blue line at (ΔpH_i_)_2/pMet↓_ = −0.08, the value for the parent disturbance, (ΔpH_i_)_2/MAc_.

**TABLE 9:** State and behavior of 61 hippocampal neurons behavior during exposure to MAc and an isolated decrease of [HCO_3_^−^]_o_ (pMet↓).

A, state:* MAc_1_	35 neurons (57%) = resistant		26 neurons (43%) = sensitive		
B, state transition:† MAc_1_ → pMet↓_2_	35 (57%) remain resistant [Q_I_]	0 (0%) become sensitive [Q_IV_]	26 (43%) become resistant [Q_II_]	0 remain sensitive [Q_III_]	
C, Behavior:‡ MAc_1_ → pMet↓_2_*					Total
Adaptation	30 (49%)	0	26 (43%)	0	56 (92%)
Consistency	5 (8%)	0	0	0	5 (8%)
Decompensation	0	0	0	0	0
D, State:† pMet↓_2_ Resistant	61 (100%)		pMet↓_2_ Sensitive	0	

*State for pulse 1: resistant neurons are to the right of the vertical dashed blue line; sensitive neurons are to the left.

†State for pulse 2, resistant neurons are above the horizontal dashed blue line; sensitive neurons are below.

‡Behaviors for pulse 1→2 transition: Adaptation (i.e., point is above the hourglass); Consistency (i.e., point is within the hourglass); Decompensation (i.e., point is below the hourglass).

Q, quadrant; Q_I_, quadrant I (neurons that are resistant during MAc_1_, and remain “resistant” during pMet↓_2_); Q_II_, quadrant II (MAc_1_ sensitive → pMet↓_2_ resistant); Q_III_, quadrant III (MAc_1_ sensitive → pMet↓_2_ sensitive); Q_IV_, quadrant IV (MAc_1_ resistant → pMet↓_2_ sensitive).

**Key points:** The population of HC, neurons used in these experiments has a greater fraction of MAc_1_-resistant cells (35 of 61 = 57% to the right of the vertical dashed blue line) than in other parts of the present paper. All of the neurons that are resistant during MAc_1_ remain so during pMet↓_2_, and all of the neurons that are sensitive during MAc_1_ shift to fulfill the MAc_2_ criteria for resistance during pMet↓_2_—that is, all the neurons are resistant during pMet↓_2_. 92% of the neurons fulfill the criteria for adaptation. Although these data are not listed in this table, MAc_1_-resistant neurons had a somewhat greater probability (32 of 35 = 91%) undergoing a paradoxical alkalinization during pMet↓_2_ than did MAc_1_-sensitive neurons (21 of 26 = 81%).

The most striking aspects of Figure 9B are that few neurons are below the *x*-axis, and none are below either the horizontal dashed blue line or the hourglass. Thus, MAc pretreatment produces a dramatic alkaline-shift of the pH_i_ response to pMet↓_2_ during the second challenge. In fact, 87% of the neurons exhibit a frank alkalinization.

The blue point lies in Q_I_ (i.e., states are both MAc_1_ and pMet↓_2_ resistant), above the hourglass (i.e., behavior is adaptation), and above the *x*-axis (i.e., pMet↓_2_ elicits a paradoxical alkalinization).

The green point lies in Q_II_ (i.e., states are MAc_1_ sensitive but pMet↓_2_ resistant), above the hourglass (i.e., behavior is adaptation), and above the *x*-axis (i.e., pMet↓_2_ elicits a paradoxical alkalinization).

The red point lies on the Q_I_ (i.e., states are both MAc_1_ and pMet↓_2_ resistant), above the hourglass (i.e., behavior is adaptation), as well as to the right of the *y*-axis and above the *x*-axis (i.e., both MAc_1_ and pMet↓_2_ elicit paradoxical alkalinizations).

The mean *d*_±_ is +0.094 (Table 2, row 7), the most strongly positive value in the present study; this value is significantly different from zero (p ≅ 2.3×10^−15^), consistent with adaptation.

#### **Δ**pH_i_ vs initial pH_i_

Figure 9C reveals correlation strengths, taking all 61 points together, of “absent” for (ΔpH_i_)_1/MAc_ vs (pH_i_)_1_, and “weak” (ΔpH_i_)_2/pMet↓_ vs (pH_i_)_2_.

#### Frequency distributions

The subset of neurons summarized Figure 9D has a mean (ΔpH_i_)_1/MAc_ of −0.07, less than the global value of −0.11 across the entire study (see Figure 1). However, far more striking is the mean (ΔpH_i_)_2/pMet↓_, which is shifted far to the right, at a positive value of +0.06. The difference is highly significant.

#### Summary of MAc-pMet**↓**

Pretreatment with MAc causes 87% of neurons to alkalinize paradoxically in response to pMet↓_2_.

## Discussion

### Historical background and comparisons

Previous work from this laboratory has shown that, in response to MAc, the cytosol of hippocampal neurons acidifies to varying extents, with some neurons undergoing relatively small pH_i_ decreases and being described as “resistant” (Bouyer et al., 2004). In a subsequent study of ten different cell types, including co-cultured mouse HC neurons and astrocytes, Salameh et al. (2014) numerically defined “MAc resistant” and “MAc sensitive”. Salameh et al. (2014) also introduced and defined the terms “adaptation,” “consistency,” and “decompensation” to describe the change in MAc-induced ΔpH_i_ values between the first and second of two MAc challenges. Salameh et al. (2017) later established that the acceleration of the Cl-HCO_3_ exchanger AE3 (a potent acid loader) plays a central role in determining the rate and extent of the MAc-induced acidification of HC neurons. Thus, we expect that the fall in [HCO_3_^−^]_o_ per se—one component of MAc—to accelerate AE3 and thereby contribute to the MAc-induced fall in pH_i_. However, we do not know whether the fall in [HCO_3_^−^]_o_, by other mechanisms (e.g., effects on other transporters or regulatory processes), contributes to the fall in pH_i_. Nor do we know whether the concomitant fall in pH_o_ per se—the other component of MAc—also contributes to the MAc-induced fall in pH_i_.

Understanding the mechanism of action of MAc is crucial for two reasons. First, several diseases can cause MAc. These include sepsis, diabetic ketoacidosis, ischemia/hypoxia (causing lactic acidosis), kidney disease (causing renal tubular acidosis), and gastrointestinal diseases (causing severe diarrhea). Second, MAc can negatively affect various organ systems, even to the extent of being life-threatening (Lim, 2007; Gennari and Weise, 2008; Raphael et al., 2016; Raphael, 2018).

Of the 235 naïve neurons exposed to MAc in the present study, ∼62% were MAc sensitive and ∼38% were resistant. This distribution is similar to the one reported by Salameh et al. (2014) but differs from our first study, in which we reported that the majority of hippocampal neurons were resistant (Bouyer et al., 2004). Part of this difference is due to the resistant/sensitive criteria later established by Salameh et al. (2014), and the rest presumably arises from the small number of neurons (14 neurons) in that first study, compared to 25 in the work by Salameh et al. (2014), and 235 in the present study.

Zhou et al. (2005) previously reported the effects of select OOE solutions on steady-state pH_i_ in rabbit proximal tubules. The present study is the first on vertebrate cells to report pH_i_ time courses during the application and removal of OOE solutions. Following up on previous work on the effects of MAc and MAc-MAc on neurons and other cells by Salameh et al. (2014), our major goal in the present study was to use OOE technology as a tool to dissect the effects of MAc into two of its major component parts (1) an isolated decrease in pH_o_ (i.e., pAc): and (2) an isolated decrease in [HCO_3_^−^]_o_ (i.e., pMet↓).

### Effects of acid-base challenges on [CO_3_^=^]_o_

As noted by Zhao et al. (2003) and Bouyer et al. (2024), the approach for creating OOE solutions does not permit independent control over CO_3_^=^ or NaCO_3_^−^. These solutes are important because, as suggested earlier (Boron and Boulpaep, 1983; Boron and Russell, 1983; Boron, 1985; Boron and Knakal, 1989; 1992), a combination of electrophysiological and modeling approaches now shows that either CO_3_^=^ or the NaCO_3_^−^ ion pair is the actual substrate of both the electrogenic Na/HCO_3_ cotransporter NBCe1 and the Na^+^-driven Cl-HCO_3_ exchanger NDCBE (Lee et al., 2023). Because both transporters play important roles in pH_i_ regulation of both neurons and astrocytes, it is instructive to consider how our experimental challenges impact [CO_3_^=^]_o_:

• During MAc in our experiments, [HCO_3_^−^]_o_ falls from 22 mM to ∼14 mM, which is ∼63% of the initial value. Simultaneously, pH_o_ falls from 7.40 to 7.20, which means that [H^+^]_o_ rises by a factor of 1/(∼63%) or ∼58%. The rise in [H^+^]_o_ produces a reciprocal fall in [CO_3_^=^]_o_. Thus, this combination of [HCO_3_^−^]_o_ and pH_o_ changes in MAc causes [CO_3_^=^]_o_ to fall to about 63 % × 63 % or ∼40% of its initial value.
• During pAc, where [CO_2_]_o_ and [HCO_3_^−^]_o_ are fixed, [H^+^]_o_ rises by ∼58% (i.e., corresponding to the same 0.2 pH_o_ decrease as in MAc), which causes a reciprocal decrease in [CO_3_^=^]_o_, which falls to ∼63% of its initial value.
• During pMet↓, where [CO_2_]_o_ and [H^+^]_o_ are fixed, [HCO_3_^−^]_o_ falls to ∼63% of its initial value (as in MAc), which causes [CO_3_^=^]_o_ to fall to ∼63% of its initial value.

In summary, pAc and pMet↓ each produce 37% decreases in [CO_3_^=^]_o_, and also [NaCO_3_^−^]_o_, whereas MAc produces a 60% decrease.

### Comparison of effects of MAc vs those of pAc + pMet↓

In this section, we ask whether the effects of MAc are merely the sum of the individual effects of pAc and pMet↓. Based on our discussion of how each challenge affects [CO_3_^=^]_o_, we expect the decrease in [CO_3_^=^]_o_ during MAc (∼60%) to be modestly less than the sum of the decreases during pAc (∼37%) and pMet↓ (∼37%).^9^

#### (1) Effects on (**Δ**pH_i_)_1_: MAc_1_ vs pAc_1_ + pMet**↓**_1_

The simplest question addresses “state”: is the effect of MAc on ΔpH_i_ in a naïve neuron merely the sum of the acidosis (i.e., pAc) and the decrease in [HCO_3_^−^]_o_ (i.e., pMet↓)? To address this query, we compare MAc_1_ (Figure 3) vs pAc_1_ (Figure 6) and pMet↓_1_ (Figure 8). The answer seems to be approximately “yes.” If we sum the ΔpH_i_ for the first challenge in each of these figures (compare Figure 3D vs Figures 6D vs; Figure 8D), we have a near-exact match:^10^

$$\begin{array}{l} {\left(\Delta {\text{pH}}_{i}\right)}_{1/\;\text{MAc}}\;\overset{?}{=}\;{\left(\Delta {\text{pH}}_{i}\right)}_{1/\text{pAc}}+{\left(\Delta {\text{pH}}_{i}\right)}_{1/\text{pMet}↓}\\ \underset{\text{Fig }3D}{\underbrace{-0.14}}\;≅\underset{-0.14}{\underbrace{\underset{\text{Fig }6D}{\underbrace{-0.10}}+\underset{\text{Fig }8D}{\underbrace{-0.04}}}} \end{array}$$ (3)

If instead, we use the global (ΔpH_i_)_1/MAc_ from Figure 1, we observe only a modest difference

$$\begin{array}{l} {\left(\Delta {\text{pH}}_{i}\right)}_{1/\text{MAc}}\;\overset{?}{=}\;{\left(\Delta {\text{pH}}_{i}\right)}_{1/\text{pAc}}+{\left(\Delta {\text{pH}}_{i}\right)}_{1/\text{pMet}↓}\\ \underset{\text{Fig }1}{\underbrace{-0.11}}\;≅\underset{-0.14}{\underbrace{\underset{\text{Fig }6D}{\underbrace{-0.10}}+\underset{\text{Fig }8D}{\underbrace{-0.04}}}} \end{array}$$ (4)

One note of caution is that, because we did not set out to perform a systematic comparison of MAc vs pAc and pMet↓, we did not routinely perform all three protocols on the same day on naïve neurons from the same cultures. Nevertheless, we are comparing data from a large number of neurons, coverslips, and cultures across a large portion of the present study.

A second note of caution is that, as noted above under “Effects of acid-base challenges on [CO_3_^=^]_o_”, the sum (Δ[CO_3_^=^]_o_)_1/pAc_ + (Δ[CO_3_^=^]_o_)_1/pMet↓_ is a somewhat larger negative number than (Δ[CO_3_^=^]_o_)_1/MAc_:

$$\begin{array}{l} {\left(\Delta {\left[{\text{CO}}_{3}^{=}\right]}_{i}\right)}_{1/\text{MAc}}\;\overset{?}{=}\;{\left(\Delta {\left[{\text{CO}}_{3}^{=}\right]}_{i}\right)}_{1/\text{pAc}}+{\left(\Delta {\left[{\text{CO}}_{3}^{=}\right]}_{i}\right)}_{1/\text{pMet}↓}\\ \underset{\text{Fig }3}{\underbrace{↓60\%}}\;\;<\;\underset{↓74\%}{\underbrace{\;\underset{\text{Fig }6}{\underbrace{↓37\%}}+\underset{\text{Fig }8}{\underbrace{↓37\%}}}} \end{array}$$ (5)

This inequality could contribute to the any imbalance in Equation 3 and Equation 4. Moreover, the three acid-base challenges presumably also lead to different changes in various cytosolic parameters (see Bouyer et al., 2024) that could contribute to ΔpH_i_ in ways that are not algebraically additive. For example, in all three disturbances, [CO_2_]_o_ is fixed, so that the induced changes in pH_i_ translate directly to changes in [HCO_3_^−^]_i_ and [CO_3_^=^]_i_, which could in turn have nonlinear effects on the kinetics of transporters.

Our conclusion from this first analysis is that for naïve neurons—cells experiencing a challenge for the first time—the whole (MAc_1_) is very nearly the sum of the parts (pAc_1_ + pMet↓_1_). We address this additivity in Bouyer et al. (2024).

#### (2) Effects of MAc_1_ on (**Δ**pH_i_)_2/MAc_ vs (**Δ**pH_i_)_2/pAc_ + (**Δ**pH_i_)_2/pMet**↓**_

Question ‘2’ is similar to ‘1’, but addresses “behavior” across twin-pulse challenges. Here we ask whether the effect of MAc_1_ on MAc_2_ (Figure 3B) is the sum of the effects of MAc_1_ on pAc_2_ (Figure 7B) and MAc_1_ on pMet↓_2_ (Figure 9B). First, examining the ΔpH_i_ values:

$$\begin{array}{l} {\left(\Delta {\text{pH}}_{i}\right)}_{1/\text{MAc}-2/\text{MAc}}\;\overset{?}{=}\;{\left(\Delta {\text{pH}}_{i}\right)}_{1/\text{MAc}-2/\text{pAc}}+{\left(\Delta {\text{pH}}_{i}\right)}_{1/\text{MAc}-2/\text{pMet}↓}\\ \underset{\text{Fig }3D}{\underbrace{-0.11}}\;\ll \underset{-0.01}{\underbrace{\underset{\text{Fig }7D}{\underbrace{-0.07}}+\underset{\text{Fig }9D}{\underbrace{+0.06}}}} \end{array}$$ (6)

In Equation 6, the three (ΔpH_i_) values refer to the mean (ΔpH_i_)_2_ in protocols in which the first challenge was always MAc_1_, but the second challenges were MAc_2_ on the left vs pAc_2_ and pMet↓_2_ on the right. Thus, observing (ΔpH_i_)_2_, we see that the effects of MAc_1_ on (ΔpH_i_)_2/pAc_ and (ΔpH_i_)_2/pMet↓_ very nearly cancel one another (i.e., −0.01), as they sum to a value that is markedly smaller than the effect of MAc_1_ on (ΔpH_i_)_2/MAc_ (i.e., −0.11).

Viewed from the perspective of *d*_±_, in the MAc-MAc protocol, *d*_±_ is +0.024 (see Table 2), compared to a *d*_±_ of +0.020 in the MAc-pAc protocol and a *d*_±_ of +0.094 in the MAc-pMet↓ protocol. Thus, for these three protocols that have in common that the first challenge is MAc, we ask in equation form:

$$\begin{array}{l} {\left(d_{\pm }\right)}_{1/\text{MAc}-2/\text{MAc}}\;\;\overset{?}{=}\;{\left(d_{\pm }\right)}_{1/\text{MAc}-2/\text{pAc}}+{\left(d_{\pm }\right)}_{1/\text{MAc}-2/\text{pMet}↓}\\ \underset{\text{Fig }3B}{\underbrace{+0.024}}\;\ll \;\underset{+0.114}{\underbrace{\underset{\text{Fig }7B}{\underbrace{+0.020}}+\underset{\text{Fig }9B}{\underbrace{+0.094}}}} \end{array}$$ (7)

In other words, with MAc_1_ as the first challenge and MAc_2_ as the second, we see a modest adaptive effect (+0.024), whereas the two component second challenges—pAc_2_ (modest adaptive effect) and pMet↓_2_ (strong adaptive effect)—produce effects that sum to an extremely strong adaptive effect (+0.114). Clearly, the whole is much less than the sum of the parts.

From this third analysis, whether we examine ΔpH_i_ or *d*_±_, we conclude that—when the first challenge is MAc_1_—the whole (MAc_2_) is very different from sum of the parts (pAc_2_ + pMet↓_2_).

Similar to what we proposed above regarding question ‘2’, we suggest that—following MAc_1_—the machinery triggered by pAc_2_ and pMet↓_2_ intersect in such a way that, in combination but not alone, the two produce a modest, net adaptation behavior. In other words, the ↓(pH_o_)_2_ and ↓([HCO_3_^−^]_o_)_2_ signals must be coincident in order to produce the physiological effect of MAc_2_. We address the issue of coincidence in Bouyer et al. (2024).

#### (3) Effects on (**Δ**pH_i_)_2/MAc_: Ac_1_ vs pAc_1_

It is instructive to compare the effects of Ac_1_ vs pAc_1_ on (ΔpH_i_)_2/MAc_ because Ac_1_ and pAc_1_ (from the perspective of pH_o_) differ only in the absence vs presence of CO_2_/HCO_3_^−^. In the Ac-MAc protocol (see Figure 4D), (ΔpH_i_)_2/MAc_ is −0.09, whereas in the pAc-MAc protocol (see Figure 6D), (ΔpH_i_)_2/MAc_ is −0.13. Although these means are not significantly different (*p* = .0982), the trend is for acidosis in the presence of CO_2_/HCO_3_^−^ to produce a more negative (ΔpH_i_)_2/MAc_, consistent with a trend towards greater decompensation.

We see an analogous but stronger pattern when we compare *d*_±_ values (see Table 2). The *d*_±_ of +0.015 for Ac-MAc (see also Figure 4B) is exactly opposite the *d*_±_ of −0.015 for pAc-MAc (see also Figure 6B). Again, although the difference in mean *d*_±_ values is not statistically significant (*p* = .0919), the trend is toward greater decompensation when the acidosis in the first challenge, occurs in the presence of CO_2_/HCO_3_^−^.

### Paradoxical effects of pMet↓

The effects of pMet↓ are truly unique. In the naïve neurons of Figure 8A and B, 19 of 52 (36%) of neurons exhibit a paradoxical pH_i_ increase—a positive (ΔpH_i_)_1/pMet↓_.

**FIGURE 10:**
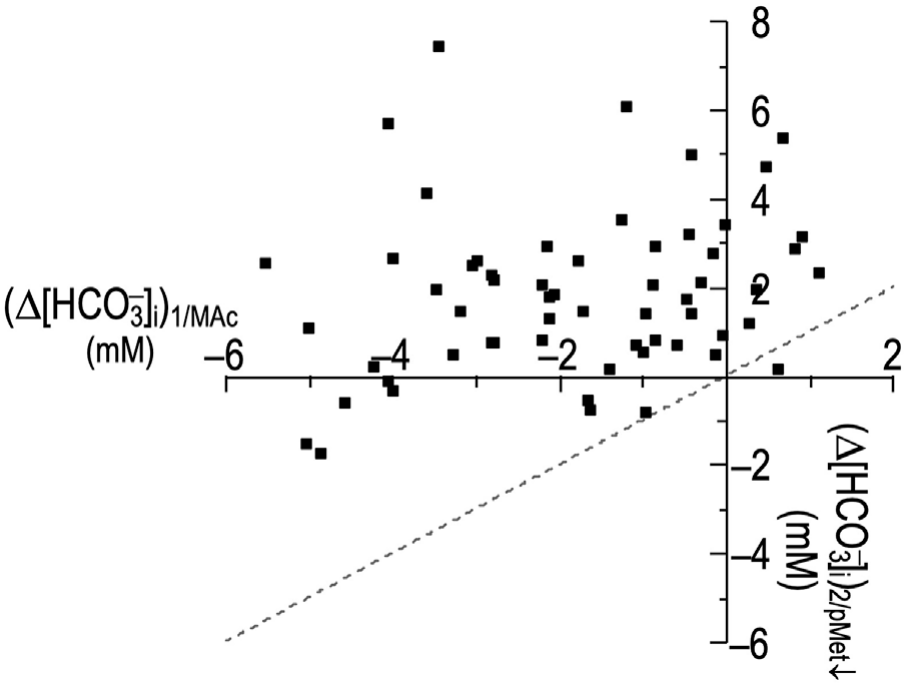
Effect of the MAc-pMet↓ protocol on computed [HCO_3_^−^]_i_. Here we plot [HCO_3_^−^]_i_ values that we computed from the same primary pH_i_ data that yielded the ΔpH_i_ data in Figure 9B, with the assumption that [CO_2_]_o_ = [CO_2_]_o_. On the abscissa is the Δ[HCO_3_^−^]_i_ during MAc_1_, and on the ordinate, the Δ[HCO_3_^−^]_i_ during pMet↓_2_. The dashed gray line represents the line of identity. Note that the pH-driven concepts of resistant/sensitive (which gave us the vertical and horizontal dashed blue lines in Figure 3B through Figure 9B) and the hourglass have no meaning in the plot we show here because the Δ[HCO_3_^−^]_i_ depends not just on ΔpH_i_ but also on the pH_i_ just before the imposition of MAc_1_.

Even more striking is the MAc-pMet↓ protocol (see Figures 9A, B), where MAc_1_ pretreatment causes the response to pMet↓_2_ to be a frank pH_i_ increase in 53 of 61 (∼87%) of the neurons. In Figure 10, we use the pH_i_ data from Figure 9B to produce a plot of (Δ[HCO_3_^−^]_i_)_1/MAc_ vs (Δ[HCO_3_^−^]_i_)_2/pMet↓_. All but one of the points lie above the LOI; that is, in 60 of 61 neurons, (Δ[HCO_3_^−^]_i_)_2/pMet↓_ > (Δ[HCO_3_^−^]_i_)_1/MAc_. Moreover, 53 of 61 neurons (the same 53 as in Figure 9B) lie above the *x*-axis. In other words, in these 87% of neurons, pMet↓_2_ (i.e., reducing [HCO_3_^−^]_o_ from 22 to ∼14 mM) causes [HCO_3_^−^]_i_ to rise. This effect is analogous to putting 61 glasses of room-temperature water into a (functioning) refrigerator and removing the glasses 5 min later, only to find that the water temperature has paradoxically risen in 53 of 61 glasses (!).

As we speculate in Bouyer et al. (2024), the paradoxical pH_i_ increase during pMet↓_1_ in many naïve neurons in Figures 8A, B—a “state”—likely reflects a novel response to the decrease in [HCO_3_^−^]_o_ *per se*, perhaps mediated by receptor protein tyrosine phosphatases γ (RPTPγ) and/or ζ (RPTPζ), both of which are expressed in mouse HC neurons (Taki et al., 2024).

The paradoxical pH_i_ increase during pMet↓_2_ of the twin MAc-pMet↓ challenge in Figures 9A, B—a “behavior”—could reflect the actions of intracellular and extracellular acid-base sensors (including the RPTPs) during MAc_1_, as well as the RPTPs during pMet↓_2_. It may be that, during a MAc-MAc protocol, the collection of all intra- and extracellular sensors acting during MAc_1_ make the neuron better able to withstand a subsequent MAc_2_. However, when pMet↓_2_ replaces MAc_2_, the lone immediate challenge to the now-prepared neuron is a decrease in [HCO_3_^−^]_o_. The unbalanced response of the hypothetical receptor to low [HCO_3_^−^]_o_ could be a massive stimulation of acid extruders (vs acid loaders), resulting in the strong and nearly uniform paradoxical pH_i_ increase.

## Summary and conclusions

• **Sensing:** Rat HC neurons can separately sense and respond to ↓(pH_o_) and ↓([HCO_3_^−^]_o_).
• **State:** In naïve HC neurons, the separate ↓(pH_o_) and ↓([HCO_3_^−^]_o_) signals summate to yield the same ΔpH_i_ as the simultaneous ↓(pH_o_)/↓([HCO_3_^−^]_o_) signals.
• **Behavior:** When HC neurons cross the boundary between two acid-base challenges, the ΔpH_i_ or *d*_±_ response is a complex event that requires coincident pAc and pMet↓ signals.

## Data Availability

The raw data supporting the conclusions of this article will be made available by the authors, without undue reservation.

## Footnotes

1 See Methods > Definitions.

2 For the neuron represented by the red record, pH_i_ at the outset of the experiment at first appears to decline and then nearly stabilize just before MAc_1_. The pH_i_ declines are initially slow but continuous throughout the MAc periods—reminiscent of AE3-KO neurons in the study by Salameh et al. (2017). They proposed that, during MAc, AE3 exchanges intracellular HCO_3_^−^ for extracellular Cl^−^, which causes (a) an initial rapid fall of pH_i_, (b) loads the cell with Cl^−^, thereby maintaining [Cl^−^]_i_, thereby (c) maximizing acid extrusion and stabilizing pH_i_ during the latter portion of the MAc. Thus, AE3 would promote a rapid but limited pH_i_ decline. A cell with low AE3 activity (or any phenotype that would lower [Cl^−^]_i_) could behave like the red neuron.

3 Note that using the quadrants to track the “state” of a neuron from MAc_1_ to MAc_2_ is not necessarily the same as using the hourglass (discussed below under #2) to define “behavior”.

4 See Methods > Definitions > Behavior.

5 The line segment between the point and the line of identity is, by definition, orthogonal to the line of identity. Because the *x*-axis scale in Figure 3B is slightly smaller than the *y*-axis scale, the gold-colored line segment is not quite orthogonal to the dashed line of identity.

6 Methods > Data analysis and statistics.

7 For an example of how to interpret this table, see the analogous presentation in the MAc-MAc section.

8 The duration of experiments is limited by the health of the neuron, which we judge by *–k*_440_.

9 In the present study, we examine only a subset of properties related to acid-base physiology. One could address our question to a far greater range of pH_i_-related properties, let alone those related to a host of other areas such as the homeostasis of other ions and electrophysiological activity.

10 In this and subsequent equations, the bolded subscripts indicate the parameters on which we are focusing.
